# HapA protease targets PAR-1/2 to modulate ERK signalling and reduce cancer cell viability

**DOI:** 10.1038/s41420-025-02691-7

**Published:** 2025-08-28

**Authors:** David Tena-Chaves, Inês Pontes-Gomes, José Ángel Palomeque, Eric Toh, Palwasha Baryalai, Gabor Kadler, Reto A. Schuepbach, Dorothea M. Heuberger, Antoni Hurtado, Sun Nyunt Wai

**Affiliations:** 1 Centro de Investigación del Cáncer, CSIC and Universidad de Salamanca, Salamanca, Spain; 2 Department of Molecular Biology, Umeå University, Umeå, Sweden; 3 The Laboratory for Molecular Infection Medicine Sweden (MIMS), Umeå University, Umeå, Sweden; 4 Umeå Centre for Microbial Research (UCMR), Umeå University, Umeå, Sweden; 5 Institute of Intensive Care Medicine, University Hospital Zurich, Zurich, Switzerland

**Keywords:** Cell signalling, Biogeochemistry

## Abstract

Recent studies reveal that *Vibrio cholerae* secretes virulence factors impacting host cell viability, though their effects on cancer cells remain unclear. However, the bacterial components and mechanisms influencing cancer cells remain largely unknown. This study investigated the effects of *V. cholerae* mutants lacking secreted proteins on carcinoma cells. We identified the hemagglutinin zinc-metalloprotease HapA as the main factor reducing cancer cell viability. HapA cleaves protease-activated receptors 1 and 2 on epithelial cancer cells at unique sites, unlike human proteases. This cleavage triggers an early and transient activation of the kinases MEK and ERK. Transient MEK and ERK activation initiates caspase 7, leading to apoptosis and reduced viability in epithelial cancer cells. Our findings underscore the significance of human protease-activated receptors as targets for bacterial protease HapA. Furthermore, we demonstrate that selective cleavage of PAR-1/2 by HapA adjusts MEK-ERK signalling dynamics, suggesting potential new avenues for the development of novel anticancer therapies. Understanding how pathogens like *V. cholerae* interact with cancer cells sheds light on potential mechanisms underlying cancer progression and suggests new therapeutic targets for cancer treatment.

## Introduction

Bacterial toxins and enzymes are increasingly recognized for their anticancer properties, influencing cellular processes such as apoptosis, differentiation, and proliferation. It has been proposed in a multitude of publications that the integration of bacterial products with existing anticancer treatments or irradiation may amplify the effectiveness of cancer therapies [1]. For example, the protein Azurin, produced by *Pseudomonas aeruginosa*, which inhibits cancer cell growth by interacting with p53 and blocking the EphB2 signalling pathway. Furthermore, peptides derived from Azurin have shown to be effective against breast cancer, melanoma, and oral squamous carcinoma cells [2–4]. Our previous work has also underscored the significant anticancer effects of the flagellar-mediated secreted protein MakA from *Vibrio cholerae* [5–8].

Microbial proteases play a substantial role in cancer therapy through induction of apoptosis in cancer cells. For instance, a study involving a protease from *Serratia marcescens* demonstrated tumour regression in BALB/c mice with solid tumours [9]. Another notable protease with considerable pharmaceutical and clinical relevance is L-asparaginase, a microbial enzyme, which has shown potential in cancer therapy [10]. Bacterial subtilisin, a serine protease, has also been found to induce apoptosis in breast cancer cells through a ubiquitin-mediated tubulin degradation pathway [11]. Further in vivo studies on the extracellular metalloprotease arazyme from *Serratia proteamaculans* have revealed its ability to inhibit metastatic murine melanoma by eliciting cross-reactive antibodies against matrix metalloprotease 8, suggesting a potential mechanism of the protease for the cytotoxic action of metastatic microbial proteases on cancer cells [12].

Proteases are enzymes that cleave peptide bonds and are categorized by their catalytic mechanism into serine, cysteine, aspartic, glutamic, and threonine proteases. Metalloproteases, in particular, perform multifaceted roles in vital physiological processes such as protein digestion, apoptosis, cell differentiation, and inflammation [13]. Proteases, as signalling enzymes, play a key role in regulating cellular and immune responses through mechanisms that include the activation of protease-activated receptors (PARs). These receptors belong to the G-protein coupled receptor (GPCR) family featuring seven transmembrane domains, an extracellular N-terminus, and an intracellular C-terminus. The cleavage of the N-terminus by proteases triggers intracellular signalling by unveiling a tethered ligand [14].

All this evidence highlights the intricate balance between microbial proteases and their host targets in maintaining tissue functionality and protection. Imbalances in this system can significantly impact intestinal health, leading to various pathophysiological conditions [15]. Notably, proteases from gut bacteria may also play a role in cancer protection. In our previous study, it was discovered that *Vibrio cholerae* possessed a protective effect on the development of epithelial cancer cells [16].

*V. cholerae*, a Gram-negative bacterium, is renowned for causing cholera, a severe diarrhoeal disease that can lead to dehydration and potentially fatal outcomes due to fluid and electrolytes imbalance [17]. Building upon our previous findings and considering the cytotoxic role of *V. cholerae*, we hypothesized that its activity might negatively affect cancer cell viability. Our investigation delves into the identification of specific secreted components from *V. cholerae* responsible for this protective effect and understanding the underlying mechanisms. In this study, we reveal the pivotal role of the metalloprotease HapA, which is released by *V. cholerae* and how it affects the PAR-MEK-ERK signalling pathway, a key player in cancer cell survival. We investigate this previously unexplored area, examining the effect of HapA on PAR-MEK-ERK signalling in cancer cells. Through this exploration, we aim to provide a more comprehensive understanding of the mechanism of HapA action in cancer cells.

## Results

### The secreted protease HapA from *V. cholerae* affects the viability of cancer cell lines

**Fig. 1:**
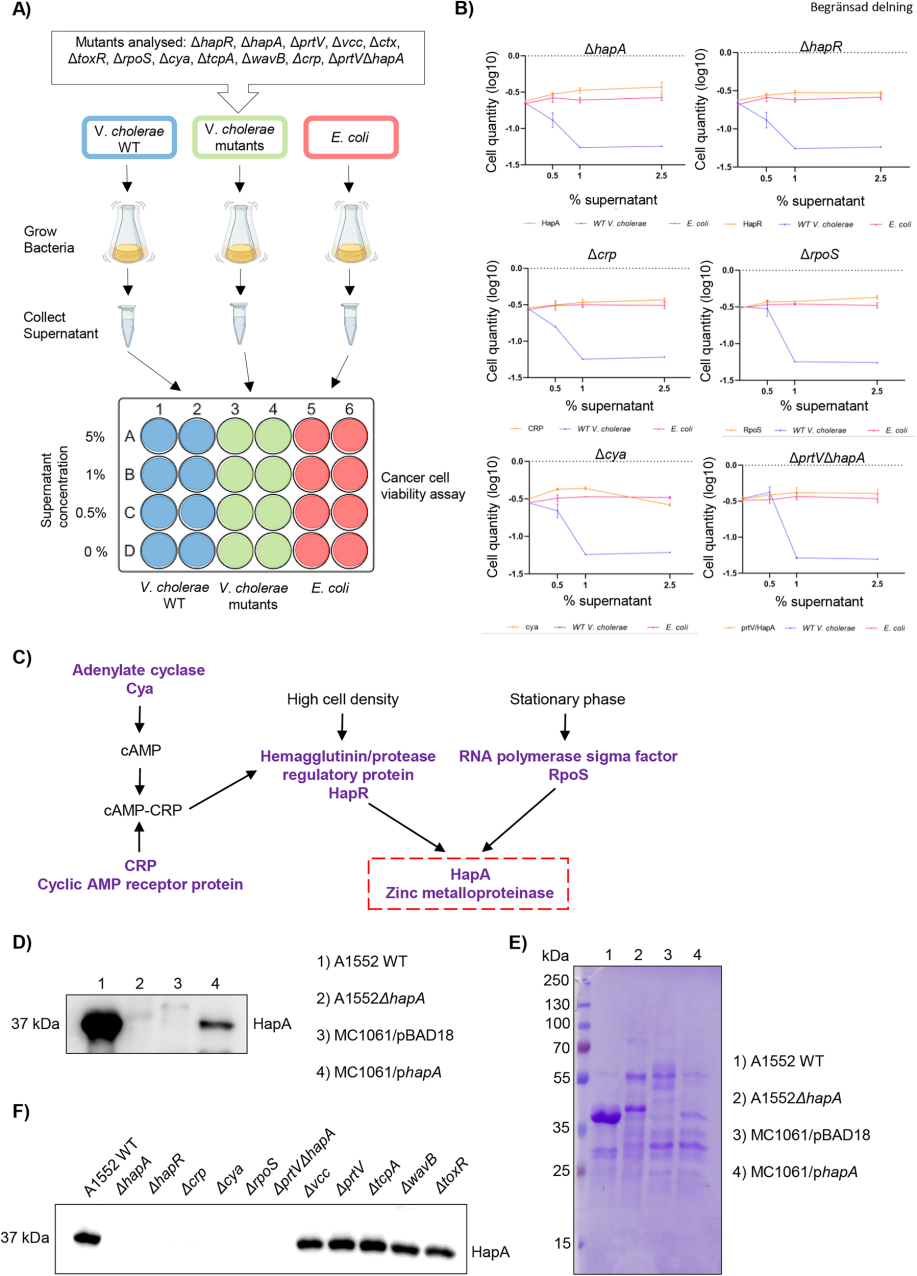
Specific secreted proteins of *V. cholerae* impact the viability of cancer cells. **A** Schematic representation of the cancer cell viability assay. **B** Viability assay in MCF-7 cells co-cultured with the supernatant from each of the 6 *V. cholerae* mutant strains with reverted effect, the wild-type *V. cholerae*, or the *E. coli* strain at concentrations of 0, 0.5, 1 and 2.5% for 24 h. Live cells were fixed and stained using crystal violet, and cell quantity was measured at 590 nm. Experiments were performed in triplicate, results were normalized applying Log10 and data are presented as mean ± SEM. (*) *p*-value < 0.05; (**) *p*-value < 0.01; (***) *p*-values < 0.001; (n.s) *p*-value > 0.05. **C** Bacterial regulatory pathway relating the proteins with a negative impact on cell viability in the assay. **D** A autodegradation HapA protein (37 KDa) detection from the supernatant of wild-type *V. cholerae*, its isogenic Δ*hapA* mutant, the *E. coli* K12 strain MC1061 habouring the pBAD18 vector, and MC1061 expressing HapA from *V. cholerae*. **E** Coomassie blue staining of the gel served as a loading control for the immunoblot shown in (**D**). **F** Western blot analysis showing HapA expression levels in supernatants isolated from A1552 wild-type and its isogenic mutants (Δ*hapA*, Δ*hapR*, Δ*crp*, Δ*cya*, Δ*rpoS*, Δ*prtV*Δ*hapA*, Δ*vcc*, Δ*prtV*, Δ*tcpA*, Δ*wavB*, and Δ*toxR*).

The primary objective of this study was to investigate the effects of proteins secreted by *V. cholerae* on human carcinoma cells. Initially, we aimed to identify specific secreted proteins in the culture media that may influence cancer cell viability. To accomplish this, we isolated culture supernatants from the wild-type *V. cholerae* strain A1552 and 12 isogenic mutants (Figs. 1A and EV1A). These mutants included those deficient in the cholera toxin, the primary virulence factor of *V. cholerae*, as well as mutants in quorum sensing regulators and the stationary-phase regulator RpoS (Table 1), following the procedures outlined in the materials and methods section. To determine the effect on cell viability, colon cancer (HCT-8) and breast cancer (MCF-7 and MDA-MB-231) cell lines were incubated with different concentrations of bacterial culture supernatants, presented as a percentage of the cell media volume (0%, 0.5%, 1%, and 5%). The viability of these cell lines was then assessed and compared with cells treated with culture supernatants from the *Escherichia col*i K12 strain MC1061, which served as a control (Fig. 1A). The data revealed that culture supernatants from the *E. coli* K12 strain MC1061 had no significant impact on cell viability at any of the examined concentrations. However, culture supernatants from the wild type *V. cholerae* strain A1552 markedly decreased the viability of both breast (Figs. 1B and EV1) and colon (Fig. EV2) cancer cells compared to control-treated cells.

**Table 1:** Bacterial strains used in this study.

Strain/Plasmid Strain	Description	Genotype/Characteristics	Source/reference
*Vibrio cholerae* A1552 WT	Wild-type strain of *V. cholerae*	O1 El Tor Inaba, RifR	[31]
A1552 Δ*hapA*	HapA protease-deficient mutant	Deletion of *hapA* gene	[32]
A1552 Δ*hapR*	Quorum-sensing regulator-deficient mutant	Deletion of *hapR* gene	[33]
A1552 Δ*crp*	cAMP receptor protein-deficient mutant	Deletion of *crp* gene	[33]
A1552 Δ*rpoS*	Sigma factor RpoS-deficient mutant	Deletion of *rpoS* gene	[34]
A1552 Δ*cya*	Adenylate cyclase enzyme-deficient mutant	Deletion of *cya* gene	[33]
A1552 Δ*tcpA*	Toxin co-regulated pilus-deficient mutant	Deletion of *tcpA* gene	[32]
A1552 Δ*ctx*	Cholera toxin-deficient mutant	Deletion of *ctx* gene	[32]
A1552 Δ*vcc*	Cytolysin-deficient mutant	Deletion of *vcc* gene	[35]
A1552 Δ*prtV*	PrtV protease-deficient mutant	Deletion of *prtV* gene	[36]
A1552 Δ*toxR*	ToxR-deficient mutant	Deletion of *toxR* gene	[34]
A1552 Δ*prtV*Δ*hapA*	Double mutant lacking HapA and PrtV proteases	Deletion of *hapA* and *prtV* genes	[32]
A1552 Δ*wavB*	LPS core oligosaccharide wavB-deficient mutant	Deletion of *wavB* gene	This study
*E. coli* MC1061	*E. coli* K12 strain	*ara*D139 Del(*ara*A-*leu*)7697 Del(*lac*)X74 *galK*16 *galE*15(GalS) lambda-e14- *mcrA*0 *relA*1 *spoT*1 *mcrB*1 *hsdR*2	[37]
MC1061/p*hapA*	*E. coli* strain expressing HapA from *V. cholerae*	*E. coli* with plasmid carrying *hapA* gene	This study
MC1061/pBAD18	*E. coli* vector control strain	*E. coli* with plasmid carrying the pBAD18 vector	This study
Plasmid			
pBAD18	Arabinose-inducible cloning vector; Ap^r^		[38]
p*hapA*	pBAD18-based HapA expression plasmid		This study

The *V. cholerae* strains that exhibited notable viability changes in MCF-7 cells with 1% of supernatant (Figs. 1B and EV1A) were: Δ*hapR* (a primary quorum-sensing regulator that affects HapA protease expression), Δ*hapA* (metalloprotease), Δ*crp* (Cyclic AMP receptor protein), Δ*rpoS* (RNA polymerase sigma factor), Δ*cya* (adenylate cyclase enzyme), Δ*ctx* (cholera toxin) and Δ*hapA*Δ*prtV* (a mutant deficient in both metalloproteases HapA and *V. cholerae* protease PrtV). Additionally, we noted that the toxic effect of HapA on MCF-7 cells varied with the dosage. Intriguingly, at higher concentrations, culture supernatants from all evaluated *V. cholerae* strains, as well as those from a non-pathogenic *E. coli* strain, exhibited toxicity (Fig. EV2A, B). Conversely, supernatants from strains deficient in other tested *V. cholerae* factors demonstrated a viability in HCT-8 and MCF-7 cells similar to that observed with the wild type *V*. *cholerae* strain across all tested concentrations (Figs. EV1A, B and EV2A, B). Furthermore, we assessed the effect of HapA on long-term cell proliferation in various cancer cell lines. Breast cancer cell lines MCF-7 and MDA-MB-231, the colon cancer cell line HCT-8, and the pancreatic cancer cell line SUIT-2 were exposed to bacterial supernatants isolated from either *V. cholerae* wild-type or the Δ*hapA* mutant strain (Fig. EV3A–D). Exposure to the wild-type bacterial supernatant inhibited cell growth in all cancer cell lines tested. In contrast, treatment with HapA-depleted supernatant reversed this inhibitory effect and resulted in a significant increase in cell proliferation between 148 and 172 hours (Fig. EV3A–D). Overall, the findings suggest that HapA plays a significant role in regulating the viability and proliferation of cancer cells in response to *V. cholerae* supernatants.

Given that *V. cholerae* releases various virulence factors into the culture supernatant, the study included the culture supernatant from an *E. coli* K12 strain harbouring the *hapA* clone to exclude the effects of other secreted factors in *V. cholerae*. Initially, we examined the presence of the HapA protein in the culture supernatants of the wild type *V. cholerae* (A1552WT), its corresponding Δ*hapA*, mutant, the *E. coli* K12 strain MC1061 with a *hapA* clone (MC1061/p*hapA*), and a vector control strain (MC1061/pBAD18) using immunoblot analysis with anti-HapA antiserum (Fig. 1D). The secretion of HapA was observed in the supernatants from *V. cholerae* A1552WT and *E. coli* MC1061/p*hapA* strains. No HapA secretion was detected in the supernatants from the A1552Δ*hapA* and MC1061/pBAD18 strains (Fig. 1D). As a control for protein loading, an SDS-PAGE gel stained with Coomassie blue was provided (Fig. 1E). Furthermore, HapA secretion was investigated in A1552WT and several isogenic mutants including Δ*hapA*, Δ*hapR*, Δ*crp*, Δ*cya*, Δ*rpoS*, Δ*prtV*Δ*hapA*, Δ*vcc*, Δ*prtV*, Δ*tcpA*, Δ*wavB*, and Δ*toxR*. HapA was detected in the supernatants of A1552WT, Δ*vcc*, Δ*prtV*, Δ*tcpA*, Δ*wavB*, Δ*toxR* strains (Fig. 1F). As expected, no HapA was observed in Δ*hapA*, Δ*hapR*, Δ*crp*, Δ*cya*, Δ*rpoS*, Δ*prtV*Δ*hapA* mutant supernatants, as these strains lack key regulatory proteins required for HapA expression (Fig. 1C and Fig. 1F). Altogether, our findings from both the wild-type *V. cholerae* and the non-pathogenic *E. coli* strain harbouring *hapA* clone, indicated that the metalloprotease HapA is responsible for affecting the viability of epithelial tumoral cells.

### Human PAR-1 and PAR-2 membrane receptors undergo non-specific cleavage by protease HapA

**Fig. 2:**
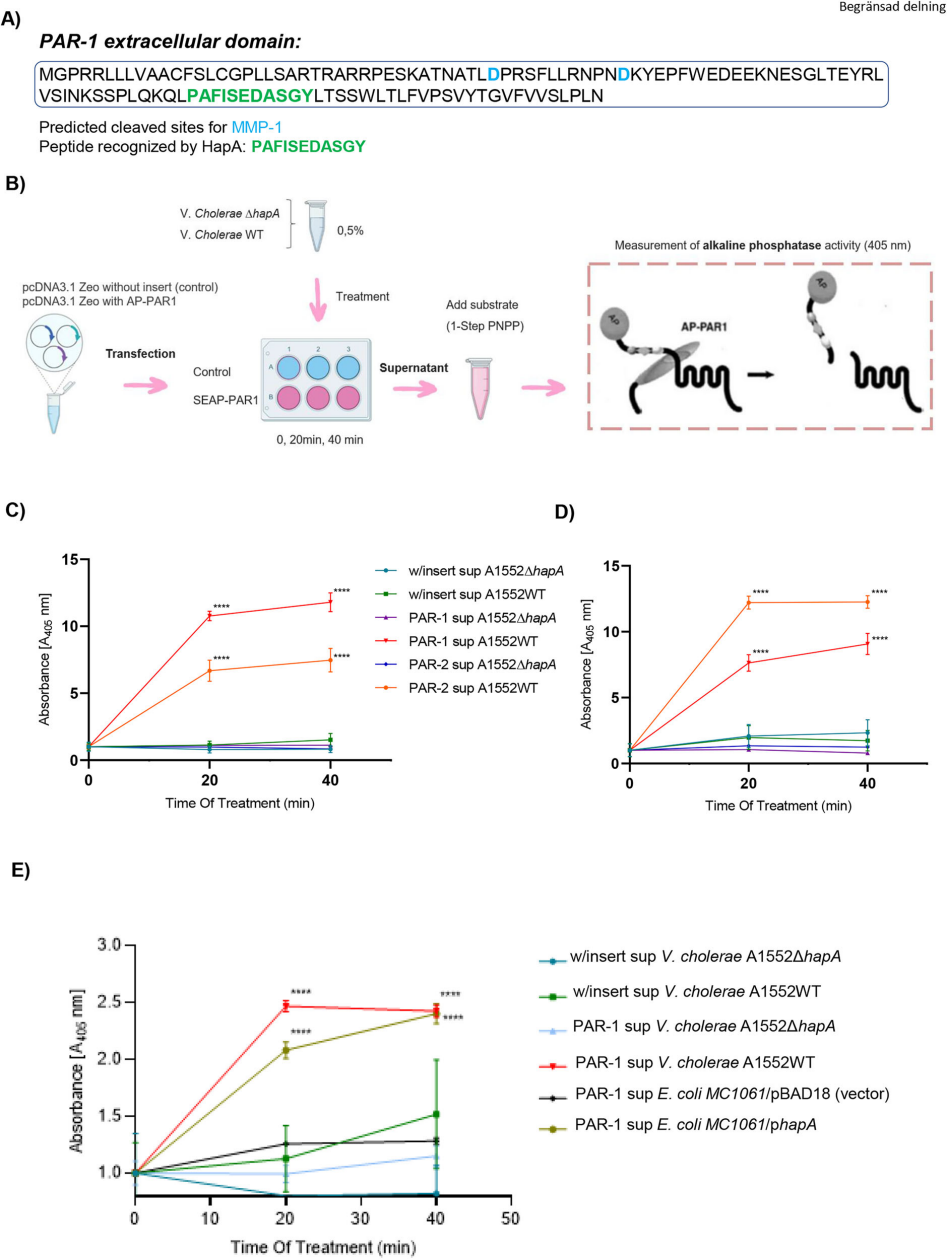
Mammalian PAR-1 and PAR-2 are cleaved by HapA. **A** In silico analysis of consensus sites for PAR-1 cleavage by eukaryotic and prokaryotic proteases. **B** PAR-1 and PAR-2 reporter constructs carrying an alkaline phosphatase (AP) on their N-terminal end releases AP upon cleavage. The quantity of alkaline phosphatase present in the medium can be quantified by incubation with the colorimetric substrate 1-Step PNPP and measured at 405 nm. **C, D** PAR-1 and PAR-2 cleavage assay in MCF-7 (**C**) and MDA-MB-231 (**D**) cells. Cells transfected with the PAR-1 or PAR-2 reporter construct or the plasmid DNA without insertion were treated with the supernatant from the wild-type *V. cholerae* or from the Δ*hapA* mutant strain at a concentration of 0.5% for 20 min and 40 min. Alkaline phosphatase activity following PAR cleavage was quantified by incubation with the colorimetric substrate 1-Step PNPP and measured at 405 nm. The experiments were performed in triplicates; results were normalized applying Log10 and data are presented as mean ± SEM. (****) *p*-value < 0.0001. **E** PAR-1 cleavage assay in MCF7 cells transfected with the PAR-1 reporter construct or the plasmid DNA without insertion were treated with the supernatants from the wild-type *V. cholerae*, Δ*hapA* mutant strain, *E.* *coli* MC1061 expressing HapA or *E. coli* MC1061 empty vector at a concentration of 0.5% for 20 min and 40 min. Alkaline phosphatase activity following PAR1 cleavage was quantified by incubation with the colorimetric substrate 1-Step PNPP and measured at 405 nm. The experiments were performed in triplicates; results were normalized applying Log10 and data are presented as mean ± SEM. (****) *p*-value < 0.0001.

Considering that HapA is a metalloprotease enzyme characterized by a conserved mechanism of action involving the cleavage of protein substrates, we hypothesized that its interaction with human epithelial cancer cells might involve the cleavage of proteins activated by such interactions. To test this hypothesis, we retrieved the peptide sequence (FISEDASGY) recognized by HapA for cleavage and conducted a search for human proteins containing this peptide. This analysis identified three potential human proteins as substrates for HapA cleavage (Fig. EV4A). Among these, Protease-Activated Receptor 1 (PAR-1), a member of the Protease-Activated Receptor (PAR) subfamily, emerged as a significant candidate. PAR-1 is a G protein-coupled receptor known to be activated by the cleavage of a portion of its extracellular domain. In silico analysis of the extracellular domain of PAR-1 revealed a consensus recognition site for HapA (Fig. 2A). Notably, the identified recognition site was located at a distance from the N-terminal domain, distinct from the known consensus site for cleavage by MMP-1 protease, a well-documented activator of PAR-1 [18], suggesting a new potential mechanism of interaction of PAR-1 with metalloproteinases.

To further investigate the effect of HapA on the activation of PARs in human cancer cells, we performed in vitro experiments to determine whether HapA facilitates the cleavage of PAR-1 and PAR-2 in cancer cell lines. These cells were transfected with a reporter construct containing PAR-1 or PAR-2 tagged with an alkaline phosphatase (AP) or, as a negative control, with a plasmid lacking the PAR gene (Fig. 2B). The mRNA encoding PAR-1 or PAR-2 was cloned to include a stop codon-truncated version of the mRNA for secreted alkaline phosphatase (AP), resulting in a chimeric PAR-1 or PAR-2 with an N-terminal AP tag [19]. The cleavage of PAR-1 or PAR-2 was monitored by measuring the release of the AP-tagged PAR-1 or PAR-2 N-terminus. After 48 hours of transfection, the cells were treated for 0, 20, and 40 min with 0.5% supernatant from either the wild-type *V. cholerae* or the Δ*hapA* mutant strain, followed by quantification of the released alkaline phosphatase indicative of PAR-1 or PAR-2 cleavage (Fig. 2B). After 20 minutes of exposure to the wild-type *V. cholerae* supernatant, both MCF-7 and MDA-MB-231 cells expressing either PAR-1-AP or PAR-2-AP demonstrated a significant increase in released alkaline phosphatase levels compared to those treated with the supernatant from the Δ*hapA* mutant strain or control cells transfected with empty vector (Fig. 2C, D). These increased levels persisted after 40 minutes of treatment with the supernatant from the wild-type *V. cholerae*, while no significant changes were observed in the cells treated with the supernatant from the Δ*hapA* mutant. Control cells transfected with the plasmid DNA lacking the PAR-1-AP or PAR-2-AP constructs showed similar levels of alkaline phosphatase at each time point in both (Fig. 2C, D). We further confirmed HapA-mediated cleavage of PAR-1 in HCT-8 and SUIT-2 cell lines (Fig. EV4B, C). To evaluate whether HapA might cleave conventional MMP-1 site at PAR-1, we repeated the experiment in MCF-7 and MDA-MB-231 cells transfected with a mutant version of PAR-1 deficient for two MMP-1 cleavage sites. The results revealed cleavage of PAR-1 when transfected cells with vectors carrying the MMP-1 mutant were exposed to *V*. *cholerae* bacterial supernatants (Fig. EV4D).

To further substantiate the selective targeting of PAR-1 by HapA, we used an *E. coli* strain genetically modified to express the HapA protease from *V. cholerae*. This approach was undertaken to delineate the effects of HapA from other virulence factors present in *V. cholerae* supernatants. Supernatants derived from both the genetically modified *E. coli*, as well as its vector control, were applied to MCF-7 cells transfected with a PAR-1-AP reporter construct. Consistent with observations in wild-type *V. cholerae*, the supernatant from the *E. coli* strain expressing the HapA protein facilitated the cleavage of PAR-1, while the supernatant from *E. coli* carrying only the vector did not produce a similar effect (Fig. 2E). The transfection efficiency and subsequent expression of the PAR-1 and PAR-2 constructs in the tested cells were verified by SDS-PAGE and Western blot analysis (Fig. EV5A–F).

These results underscore the role of the *V. cholerae* HapA metalloprotease in targeting the PAR-1 and PAR-2 receptors to a different cleavage site previously identified by mammalian metalloproteases. This conclusion is drawn from the observation that cells exposed to supernatant from wild-type *V. cholerae* showed an increased release of alkaline phosphatase into the medium, a marker of PAR-1 and PAR-2 cleavage, in contrast to cells treated with supernatant from a HapA-deficient mutant strain.

### HapA induces rapid effects on cell viability via ERK phosphorylation in cells expressing PAR-1 and PAR-2

**Fig. 3:**
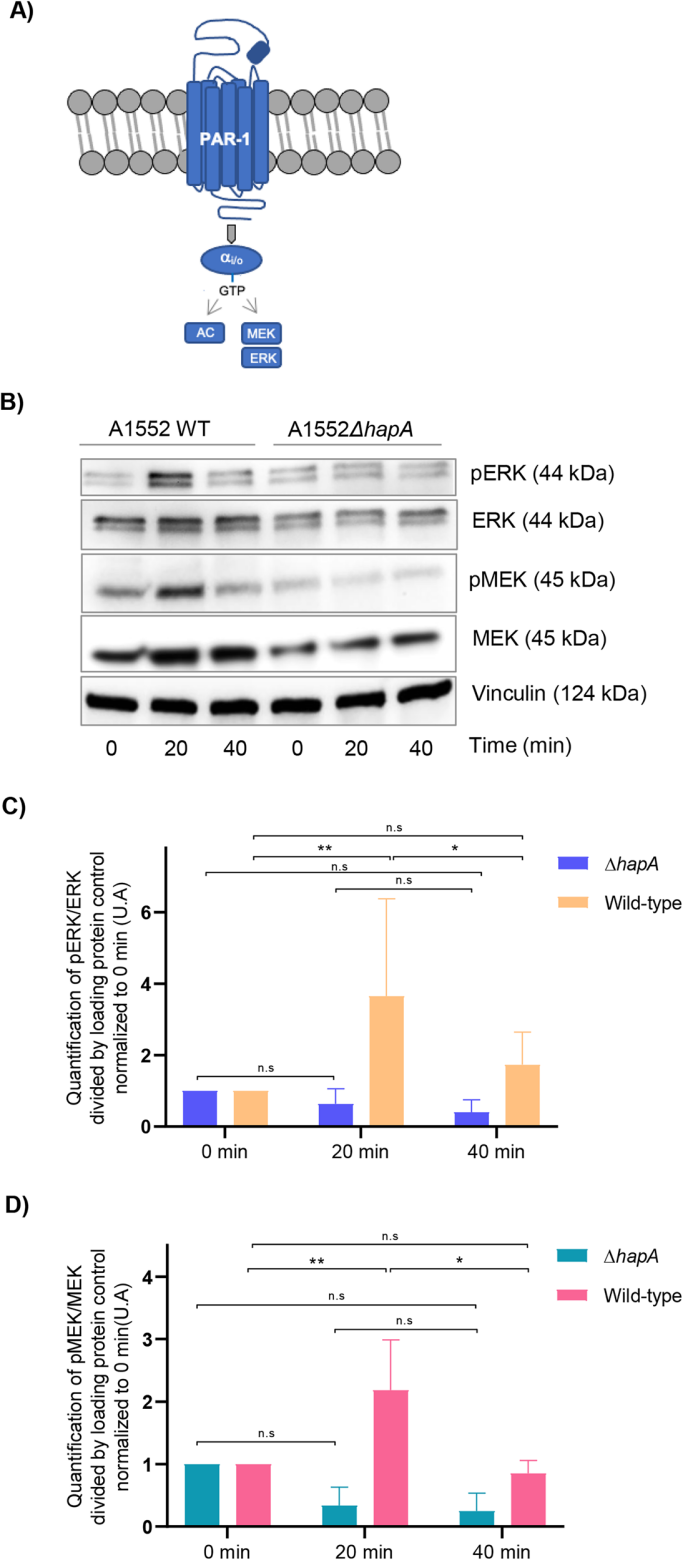
HapA induces MEK and ERK phosphorylation via PAR1. **A** Schematic illustration of PAR-1 activation of MEK/ERK signalling pathway. PAR-1 activation induces conformational changes in its transmembrane domains, which favour the interaction of the receptor with heterotrimeric G proteins. In its inactive state, the Gα_i/o_ subunit is bound to guanosine diphosphate (GDP). Activated GPCRs triggers a conformational change in the Gα_i/o_ subunit, promoting the exchange of GDP for GTP. This nucleotide exchange results in dissociation of the Gα_i/o_ subunit from the βγ complex. Coupling to Gα_i/o_ inhibits adenylate cyclase (AC), suppressing the formation of c-AMP, and thereby activating MAPK signalling. **B** MCF-7 cells were transfected with the PAR-1 reporter construct and were treated with the supernatant from the wild-type *V. cholerae* (A1552 WT) or from the Δ*hapA* mutant (A1552 Δ*hapA*) strain at a concentration of 0.5% for 0, 20 and 40 minutes. Cells were collected, and protein extracts were analysed by SDS-PAGE and western blot for the expression of ERK, p-ERK, MEK and p-MEK. Vinculin was used as a loading control. The bottom part of the figure provides quantitative analysis of the experiment in MCF-7 cells with visualized bands for ERK, p-ERK (**C**), MEK and p-MEK (**D**) normalized by loading control. The experiments were performed in triplicates. A representative experiment is shown. (*) *p*-value < 0.05; (**) *p*-value < 0.01; (n.s) *p*-value > 0.05.

Our investigation aimed to elucidate the mechanistic basis through which HapA influences the viability of cancer cells. Given the established correlation between PAR-1/2 expression and the progression of solid tumours—encompassing aspects such as tumorigenesis, invasion, metastasis, and angiogenesis—we suggested that the reduction in cell viability observed following exposure to HapA could be attributed to the disruption of downstream signalling pathways mediated by PAR-1/2. This disruption would potentially lead to a suppression of cellular proliferation and survival. To explore this, our study specifically focused on the modulation of the MEK-ERK signalling pathway by HapA treatment, considering the MER-ERK signalling pathway to play a pivotal role as a downstream effector activated by PAR-1 (Fig. 3A) and PAR-2 (Fig. EV7A) receptors [19].

**Fig. 4:**
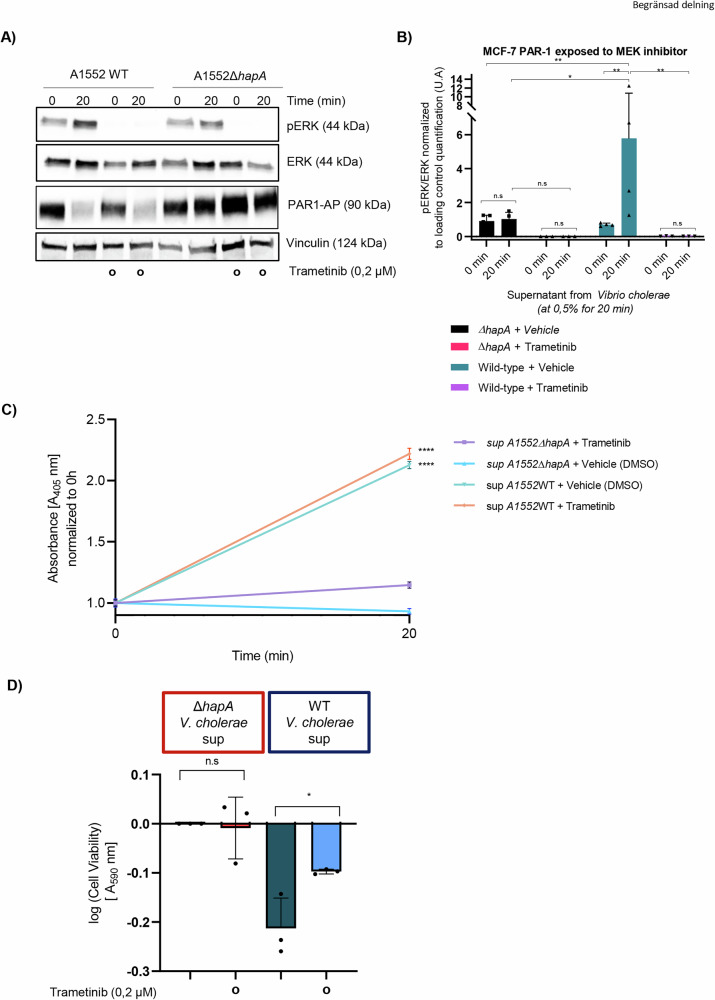
Inhibiting ERK phosphorylation mitigates the suppression of cell viability. **A** MCF-7 cells were transfected with PAR-1 reporter construct and treated with the supernatant from the wild-type *V*. *cholerae* or HapA mutant strain at a concentration of 0,5% for 0 min and 20 min, in presence of either vehicle (DMSO) or trametinib (0,2 μM). Cells were collected, and protein extracts were analyzed by SDS-PAGE electrophoresis and western blot techniques for the levels of phospho-ERK, ERK and PAR-1. Vinculin was used as a loading control. (**B**) The plot provides quantitative analysis of the experiment in (**A**), in MCF-7 cells with visualized bands for ERK, p-ERK. (*) *p*-value < 0.05; (**) *p*-value < 0.01; (n.s) *p*-value > 0.05. (**C**) PAR1 cleavage assay of MCF-7 cells transfected as in (**A**) and cultured with the supernatant from the wild-type *V*. *cholerae* or HapA mutant strain at a concentration of 0,5% for 0 min and 20 min, in the presence of either vehicle (DMSO) or trametinib (0,2 μM). Alkaline phosphatase activity following PAR-1 cleavage was quantified by incubation with the colorimetric substrate 1-Step PNPP and measured at 405 nm. Data are presented as mean ± SEM from triplicate experiments whereby (****) *p*-value < 0.0001. **D** Viability assay in MCF-7 cells co-cultured with the supernatant from wild-type *V*. *cholerae* or HapA mutant strain, in the presence and absence of trametinib (0,2 μM) for 20 min. Subsequently, both bacterial supernatant and trametinib were removed, and cells were cultured in fresh media for 24 h prior fixation and staining of cells using crystal violet. Cell viability was quantified by measuring absorbance of crystal violet at 590 nm. The experiments were performed in triplicates, results were normalized, and data are presented as mean ± SEM. (*) *p*-value < 0.05; (n.s) *p*-value > 0.05.

To test our hypothesis, all the cell lines tested in the study were engineered to express PAR-1-AP, and then incubated with supernatants from the wild-type *V. cholerae* or the Δ*hapA* mutant strain at a concentration of 0.5% for 0, 20, and 40 min. Protein extracts from both cell lines were subjected to SDS-PAGE gel electrophoresis and western blot analysis to assess the levels of total and phosphorylated ERK 1/2 and MEK (Figs. 3B–D and EV6A–I). Notably, in all the cell lines, an increase in phosphorylated ERK and MEK levels was observed after 20 minutes when treated with the supernatant from the wild-type *V. cholerae*, in contrast to those treated with the supernatant from the Δ*hapA V. cholerae* strain. Additionally, we confirmed ERK activation after 20 min in PAR-2 expressing MCF-7 and MDA-MB-231 cancer cells treated with wild-type *V. cholerae* supernatant, whereas no activation was observed in cells treated with supernatant from the Δ*hapA* strain (Fig. EV7B–E). Contrary to our initial expectations, this result indicated that the HapA-containing supernatant immediately activated the MEK-ERK signalling pathway. Nevertheless, the activation of MEK-ERK was not sustained, as evidenced by the return MEK-ERK phosphorylation to its baseline levels after 40 minutes of exposure. Subsequently, our investigation focused on determining whether the observed reduction in cell viability following PAR-1 activation was mediated by a swift and substantial activation of the MEK/ERK signalling pathway. The application of trametinib, a MEK inhibitor, significantly reduced ERK phosphorylation (Fig. 4A, B) yet had no noticeable effect on the cleavage process of PAR-1 (Fig. 4C). Importantly, trametinib treatment mitigated the adverse effect on cell viability in MCF-7 cells subjected to supernatant from wild-type *V. cholerae*, whereas its effect was minimal on cells exposed to the supernatant from *V. cholerae* strain lacking HapA (Fig. 4D). These findings suggest a critical involvement of the MEK/ERK signalling pathway in the PAR-1 and PAR-2 mediated regulation of cell viability, particularly in the cellular response to virulence factors from *V. cholerae*.

**Fig. 5:**
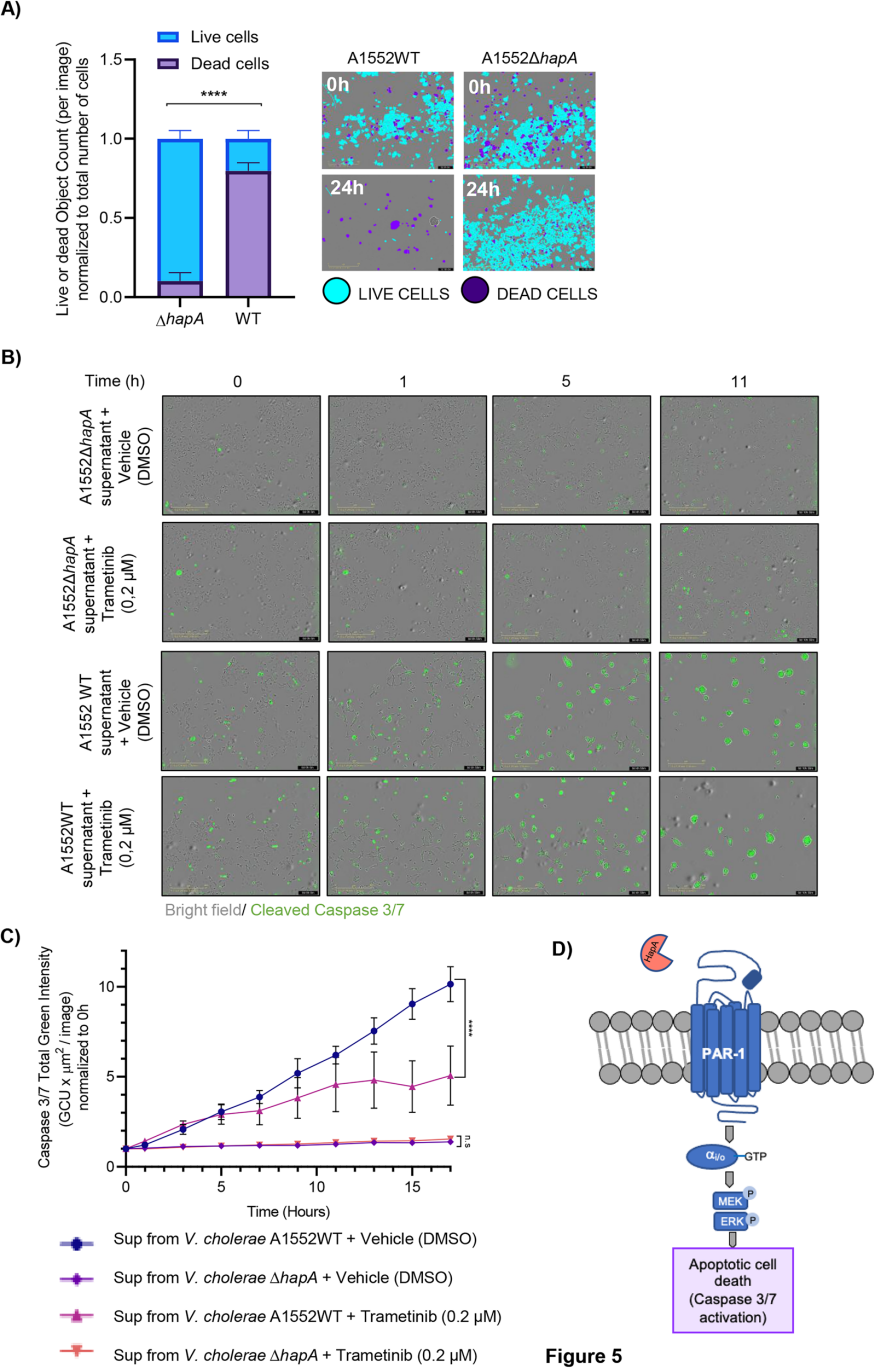
HapA triggers apoptosis in MCF-7 cells. **A** Quantification of live and dead MCF-7 cell percentages using the Incucyte® Artificial Intelligence Cell Health Analysis Module with 10X objective, 24 h after a 20-min incubation with 0.5% supernatant from either wild-type *V. cholerae* or the ΔhapA mutant strain. (**B**) Images of Caspase 3/7 Activity in MCF-7 cells treated with 0,5% of supernatant from wild-type *V*. *cholerae* or the HapA mutant strain, in the presence and absence of trametinib (0,2 μM) for 20 min. The cells were mixed with 1:1000 Caspase 3/7 Green Dye, and the plate was inserted in the Incucyte® Live Cell Imager system with 10X objective. Images were captured every hour. **C** Graph that represents total integrated Caspase 3/7 activity measured by emission of fluorescence at 530 nm from (**B**). The experiments were performed in triplicates. A representative experiment is shown. Results were normalized, and data are presented as mean ± SEM. (****) *p*-value < 0.0001; (n.s) *p*-value > 0.05. **D** Model of PAR-1 activation and downstream effects on cancer cells by HapA protease.

Taken together, our findings indicate that the HapA protease from *V. cholerae* bacteria triggers the activation of mammalian PAR-1/2 receptors, leading to a reduction in cell viability. Observing the swift impact of HapA (notably within 1–4 h), we suggested that the activation of PAR-1/2 might initiate an apoptotic cascade. To investigate this hypothesis, we first measured the number of live and apoptotic cells in MCF-7, MDA-MB-231, HCT8 and SUIT-2 cells after 20 minutes of exposure to supernatants from either wild-type *V. cholerae* or its HapA-deficient variant. Using the Incucyte® Live-Cell Imaging System (Sartorius, Essen Biosciences, Ann Arbor, MI), equipped with an artificial intelligence module, we observed that HapA caused a significant increase in death cells after 24 h across all tested cancer cell lines (Figs. 5A and EV8A–C). Furthermore, caspase-3/7 activity, a hallmark of apoptosis, was assessed in MCF-7 cells exposed to supernatants from wild-type and HapA mutant bacteria. Exposure to wild-type supernatant triggered rapid caspase-3/7 activation, detectable after just one hour of exposure (Figs. 5B, C and EV9). Treatment with trametinib significantly reduced caspase-3/7 activation in cells exposed to HapA-containing supernatant, compared to cells without trametinib treatment or those exposed to Δ*hapA* bacterial supernatant (Figs. 5B, C and EV9). This effect was observed following an 11-h trametinib pre-treatment (Figs. 5B, C and EV9). Western blot analysis confirmed caspase-7 activation in MCF-7 and MDA-MB-231 cells treated with wild-type supernatant, while no activation was observed in cells exposed to supernatants lacking HapA **(**Fig. EV8D, E). Together, our data suggest that HapA cleavage/activation of PAR1/PAR 2 receptors lead to caspase 3/7 mediated-apoptotic cell death (Fig. 5D).

## Discussion

Our research represents a pioneering investigation into the complex interplay between a virulence factor secreted by *Vibrio cholerae* and its significant impact on the viability of human epithelial carcinoma cells. Building upon the hypothesis that certain bacterial virulence factors may influence cancer cell viability, our study provides novel insights into potential strategies for cancer treatment as it has been proposed to other pathogenic bacteria [1, 8]. Our primary objective focused on analysing the culture supernatant of *V. cholerae* strain A1552 and its isogenic mutants to identify components that markedly affect the viability of colon and breast cancer cell lines. Our findings indicate that specific proteins secreted by *V. cholerae* indeed exert a notable influence on the viability of epithelial cancer cell lines, suggesting that the impact pathogenic bacteria have on cell viability extends beyond colon epithelial cells.

Our analysis identified HapA as a primary secreted protein impacting cancer cell viability. This extracellular zinc-metalloprotease is crucial in *V. cholerae* pathogenicity, being secreted into the extracellular environment during infection. In the absence of cholera toxin genes, HapA may cause a more inflammatory form of diarrhoea by decreasing transcellular epithelial resistance (TER) and eliciting localized immune responses [20]. Furthermore, bacterial adherence and colonization are enhanced when the *hapA* gene is mutated, and HapA reduces intestinal mucosa viscosity to facilitate bacterial movement toward epithelial cells [21–23]. The expression of HapA, positively regulated by the quorum-sensing regulator HapR, aids in the dissemination of pathogens from the initial infection site to uninfected locations [24–26]. While HapA is an important factor in *V. cholerae* pathogenesis, detailed analysis of its modulation of host cell signalling pathways is still in its early stages. In this work, we propose that HapA may function as an effector in *V. cholerae*, influencing cancer cell viability. Our findings not only contribute to our understanding of *V. cholerae* cell viability effects but also sheds light on potential novel mechanisms underlying the interaction of the pathogenic bacteria with host cells in the context of cancer. Our work identifies human Protease-Activated Receptors (PARs), particularly PAR-1 and PAR-2, as key mediators of *V. cholerae* protease, HapA’s effect on epithelial cancer cells. PAR receptors play pivotal roles in various physiological and pathological processes, including inflammation, coagulation, and cancer progression. They are specifically cleaved and irreversibly activated by various endogenous and exogenous proteases, with proteolytic cleavage involving conformational changes and altered affinity for intracellular G proteins [19]. Contrary to canonical activation involving proteases such as MMP-1, our findings indicate that HapA does not cleave the canonical N-terminal peptides of the PAR-1 receptor. This lack of proteolytic specificity observed suggests that HapA can alter the pro-proliferative function of PAR by selectively targeting non-conventional metalloproteases peptide sequences within the extracellular domain. Our experimental evidence identifies a peptide sequence recognized by HapA in human proteins —specifically within PAR-1— provides compelling support for this hypothesis. Previous studies have shown that the HapA protease from *V. cholerae* cleaves both Cholera toxin (CT) and heat-labile enterotoxin (LT) from *E. coli* at distinct sites [27, 28]. For CT, the primary cleavage occurred at the Ser194-Met195 junction, with an additional site at Ser193-Ser194. In contrast, LT-A was cleaved between Thr193 and Ile194, as confirmed by N-terminal sequencing of the A2 fragment, which began with Ile194 following treatment [28]. These findings indicate that HapA possesses broad target specificity, enabling it to cleave different proteins at various sites based on general structural characteristics rather than strict sequence motifs. Future research should aim to identify the HapA cleavage site of PAR.

Further exploration of the mechanisms through which the HapA protease from *V. cholerae* impacts cancer cell viability focuses on its downstream signalling effectors of PAR-1 and PAR-2 receptors. Our hypothesis posits that the influence of HapA on cell viability is linked to downstream signalling pathways, particularly the MEK-ERK pathway activated by PAR receptors. Our findings reveal that exposure to HapA induces a transient increase in MEK and ERK phosphorylation. Moreover, inhibiting the MEK/ERK pathway mitigates the reduction in cell viability induced by HapA, indicating the role of this pathway in the mechanism of HapA action. We propose that the rapid activation of MEK-ERK by PAR/HapA triggers an apoptotic response, supported by caspase 3/7 activation, connecting HapA exposure to the initiation of apoptosis in cancer cells (Fig. 5D). The pro-apoptotic function of the Ras/Raf/ERK pathway is well-documented in apoptosis induced by DNA-damaging agents or various antitumor compounds [29]. Additionally, ERK activity has been associated with classical markers of apoptosis execution, such as effector caspase-3 activation [29], aligning with our findings that HapA induces early activation of caspases. In this regard, a study by Ray and Pal [30] demonstrated that HapA exposition for 24 h elevates cellular ROS levels and initiates the apoptotic pathway via PAR-1/p38 signalling activation, resulting in reduced tumour growth of cancer cells. In our current work, we demonstrate that human PAR1/2 trigger an early and transient MEK-ERK activation to initiative the apoptotic pathway. Considering that MEK inhibition substantially but not completely reverses the observed cell viability and apoptotic effects induced by HapA, we propose that both pathways may coexist in inducing apoptosis in human epithelial cells.

In summary, our findings uncover that HapA, a bacterial protease derived from *V. cholerae*, possesses the capability to cleave human PAR receptors leading to MEK-ERK phosphorylation, and introduces a novel perspective on how bacterial factors may influence the behaviour of human cancer cells. This interaction holds the potential to induce alterations in cancer cell growth, mobility, and survival, offering a distinctive avenue compared to traditional pathways, such as those activated by MMP1. The significance of this discovery is profound, as it opens new possibilities for understanding the complex interactions between pathogenic bacteria and host cells.

## Methods

### Cell lines and cultural conditions

MDA-MB-231 and MCF-7 human breast adenocarcinoma cells, obtained from ATCC (Manassas, VA) were routinely cultured in DMEM medium (1X, Gibco) with 10% heat-inactivated bovine serum FBS supplementation and 1% of penicillin streptomycin, 1% GlutaMAX and 1% Sodium Pyruvate, and grown at 37 °C in a humidified atmosphere containing 5% CO_2_. Human ileocecal colorectal adenocarcinoma cells (HCT-8) and pancreatic cell line SUIT-2, also obtained from ATCC were cultured in RPMI 1640 (Gibco), 10% FBS supplementation, with 1% of Penicillin Streptomycin, 1% GlutaMAX and 1% Sodium Pyruvate and incubated at 37 °C in a humid 5% CO_2_ atmosphere.

### Bacterial strains

All bacterial strains, plasmids and primers used in this study are listed in Tables 1 and 2. Competent *E. coli* was used for transformation and plasmid amplification.

**Table 2:** Primers used in this study.

Name	Sequence 5’→ 3’	Restriction site	For construction of
VC0224-A	CGCTCTAGACAAGGCTGGCAAGTGATCT	*XbaI*	Δ*wavB*
VC0224-B	CCCATCCACTATAAACTAACAGACAGCAAGCGTTGGTTTACTCA		Δ*wavB*
VC0224-C	TGTTAGTTTATAGTGGATGGGGACGAGCACTACAAACGCT		Δ*wavB*
VC0224-D	CGCTCTAGACACAGGAGTGATGGATGAG	*SacI*	Δ*wavB*

### Cloning and expression of the *hapA* gene in *E. coli*

The coding DNA sequences of the *hapA* gene were PCR amplified from A1552 genomic DNA and subsequently cloned into the plasmid pBAD18 at the *SacI*/*XbaI* restriction sites. Positive clones were confirmed via sequencing. The bacterial strains *E. coli* MC1061/pBAD18 and *E. coli* MC1061/p*hapA*^+^ were created by introducing the expression vector pBAD18 without an insert (serving as a negative control) and the *hapA*^+^ pBAD18 plasmid construct, respectively, into the *E. coli* MC1061 wild-type strain through electroporation. Both strains were cultured at 37 °C in LB broth supplemented with 100 µg/mL carbenicillin, and gene expression was induced by the addition of 0.1% l-arabinose.

### Preparation of culture supernatants for cell treatment

Bacterial cultures were incubated at 37 °C in Luria–Bertani (LB) broth for 16 h. Following incubation, supernatants were obtained by centrifugation at 14,000 × *g* for 5 minutes at 4°C. These supernatants were subsequently filtered through a 0.22 μm PVDF syringe filter (Millipore, USA) for sterilization and then stored at −20°C for subsequent analyses.

### Preparation of samples for SDS-PAGE and immunoblot analysis of bacterial supernatants

Bacterial strains were cultured at 37°C in Luria–Bertani (LB) broth for 16 hours and followed by subculture the next day until reaching an OD_600_ of 2.0. After incubation, supernatants were collected by centrifugation at 14,000 × *g* for 5 minutes at 4 °C. Supernatants from the wild-type *V*. *cholerae* A1552 strain, its isogenic mutants (Δ*hapA*, Δ*hapR*, Δ*crp*, Δ*cya*, Δ*rpoS*, Δ*prtV*Δ*hapA*, Δ*vcc*, Δ*prtV*, Δ*tcpA*, Δ*wavB*, and Δ*toxR*), *E. coli* K12 strain MC1061 carrying a *hapA* clone (MC1061/p*hapA*), and the vector control strain (MC1061/pBAD18) were subjected to trichloroacetic acid (TCA) precipitation. This process included sterile filtration through a 0.22 μm PVDF syringe filter (Millipore, USA) followed by incubation with 10% (w/v) TCA on ice for 30 min. Subsequently, the samples were centrifuged at 21,500 × *g* for 15 min at 4 °C to pellet the TCA-precipitated proteins. The resulting protein pellets were dissolved in 1x SDS sample buffer and heated for 10 minutes. SDS-PAGE was employed to separate the protein samples, which were then subjected to immunoblot analysis using rabbit anti-HapA antiserum (Agrisera AB, Sweden). Additionally, Coomassie blue staining was applied to visualize protein bands resolved by SDS-PAGE.

### Cell proliferation assay

MCF-7 and MDA-MB-231 cells were seeded in 24-well plates (Nuclon^TM^ Delta Surface, Thermo Fisher Scientific) at densities of 1.5 × 10^5^ cells/well and 7.5 × 10^4^ cells/well, respectively, in a growth medium and incubated at 37 °C in 5% CO_2_. After 24 h, the medium was replaced, and the cells were treated with the supernatants, either wild-type *V. cholerae*, its 12 different isogenic mutants, or *E*. *coli* harbouring either the vector control or p*hapA* clone, in DMEM medium without FBS supplementation for 20-40 min. Subsequently, the medium was removed, and the cells were fixed using 2% formaldehyde (PanReac, AppliChem, ITW Reagents) in PBS, followed by staining with 0.25 mg/ml of crystal violet (Sigma-Aldrich) in diluted distilled water. The crystal violet stain (ACS reagent, ≥90.0% anhydrous basis, Sigma-Aldrich) was then solubilized using 10% (v/v) acetic acid (Sulpeco, Analytical Products) in distilled water. The quantity of cells was assessed by measuring the absorbance at 590 nm using a microplate reader (Infinite 200 PRO, Tecan). For the inhibitor experiment, Trametinib (Selleckchem, GSK1120212) at 0.2 μM was added during 20 minutes in combination with supernatants from *V. cholerae*. DMSO was used as a vehicle. Then, it was replaced with media without serum for 24 h prior to fixation and staining with crystal violet.

### IncuCyte® live cell imager proliferation and live/dead cell detection

A proliferation assay was performed over 6–7 days using time-course imaging with the Incucyte® SX5 Live-Cell Analysis System (2024B). HCT-8, SUIT-2 and MDA-MB-231 cells were seeded at a density of 5000 cells per well in 48-well plates, meanwhile for MCF-7 with a density of 7000 cells per well were seeded. They were initially treated for 20 minutes with 0.5% bacterial supernatant (diluted in DMEM medium) derived from either wild-type *V. cholerae* or the Δ*hapA* mutant. After treatment, the medium was replaced with DMEM supplemented with 5% FBS and plate incubated for 24 hours. A second round of treatment with the respective bacterial supernatants was applied the following day. After 24 h, 5% FBS was added, and the cells were left in culture until day 6–7. Images were acquired once daily using a 10X objective, and nine fields per well were captured. Cell health parameters—including live and dead cell counts—were analyzed using the Incucyte Artificial Intelligence-based object counting module (BA-04871). Parameters settings were: (1) segmentation sensitivity of 5 (Background/cells classification) and (2) score threshold of live/dead classification equal to 0.15. Data were normalized to the initial time point (*T* = 0 h) and are presented as mean ± SEM. Statistical significance was assessed using GraphPad Prism 8.0. (****) *p*-value < 0.0001.

### SDS-PAGE and western blotting

All the cell lines were seeded in 100 mm petri dishes (Falcon®, Corning), as described previously. After 24 h, the medium was removed, and the cells were treated with the supernatant from either the wild type or the Δ*hapA* mutant *V. cholerae* strains for 20 min and 40 min in DMEM medium without FBS supplementation. Following treatment, the medium was removed, and cells were collected and lysed using RIPA lysis buffer (25 mM Tris-HCl [pH 7,6], 150 mM NaCl, 1% NP-40, 1%Triton X-100, 1% Na-deoxycholate, 0.1% SDS, 1 mM NaF, 1 mM Na_3_VO_4_, 1 mM ß-glycerophosphate. Added from fresh, proteinase & phosphate inhibitor cocktail (Roche, 05892970001)). Protein quantification was performed using the Bradford protein assay (Bio-rad), according to the manufacturer’s protocol. Equal amounts of protein, diluted in LDS sample buffer (4X) (Noves®, life technologies^TM^) with 10% Dithiothreitol (DTT) (Roche), were subjected to gel electrophoresis on a Mini-PROTEAN TGX Precast polyacrylamide gel, 4–20% (Bio-rad), and then transferred onto a nitrocellulose membrane using the dry iBlot2 transfer (life technologies^TM^). The membranes were incubated with primary and secondary antibodies, and protein bands were visualised using chemiluminescent peroxidase solutions 1 and 2 (SuperSignal^TM^ West Thermo Scientific). Chemiluminescence was detected using an iBright Imaging System. The primary antibodies used were anti-PAR-1 (1:1000, Merck Millipore, MABF244), anti-PAR-2 (1:1000, Merck Millipore, MABF243), anti-p44/42 MAPK (1:1000, Cell Signaling, 20G11), anti-phospho-p44/42 MAPK Thr202/Tyr204 (1:1000, Cell Signaling, 20G11), MEK (1:1000, Cell Signaling, 47E6), anti phospho-MEK Ser217/221 (1:1000, Cell Signaling, 41G9) and anti-Caspase 7 (1:500, Cell Signaling, #9492). Anti-GAPDH (1:5000, Cell Signaling,14C10) and anti-Vinculin (1:5000, Merck, V9131) were used as loading control. Secondary antibodies include anti-mouse IgG (1:5000, Cell Signaling), anti-rabbit IgG (1:1000, Cell Signaling).

### Transformation and plasmid DNA isolation

The PAR-1 reporter construct (pcDNA3.1/Zeo+) carries a PAR-1 gene and the PAR-2 reporter construct carries a PAR-2 gene respectively fused with a N-terminal alkaline phosphatase (AP), whereas the control plasmid DNA lacks the PAR-AP insertion as published [19].

Plasmid DNA was transformed and amplified in competent *E*. *coli* cells. The plasmid DNA was then isolated using the Plasmid DNA midi kit (QIAGEN), according to the manufacturer’s instructions, and the final DNA concentration was measured using a NanoDrop 1000.

### Transfection and PAR1/2 cleavage assay

To generate MDA-MB-231 and MCF-7 cells expressing the DNA plasmids, transfection was carried out using Lipofectamine 3000 (Thermo Fisher Scientific), following the manufacturer’s instructions. After 48 h of transfection, cells were treated with supernatants from either the wild-type *V*. *cholerae* or its isogenic Δ*hapA* mutant, at a concentration of 0.5% in DMEM medium without FBS, for 20 min and 40 min. Afterward, supernatants were removed and filtered through a Polyvinylidene difluoride (PVDF) membrane filter (pore size, 0.45 μm). Alkaline phosphatase activity was determined by incubating with the colorimetric substrate p-nitrophenyl phosphate (1-Step PNPP, Thermo Fisher Scientific) for 30 min and measuring the absorbance at 405 nm using a microplate reader (Infinite 200 PRO, Tecan).

### IncuCyte® live cell imager caspase 3/7 assay

Cleavage of caspase 3/7 was assessed using the Incucyte® Live Cell Imager system (Sartorius, Essen Biosciences, Ann Arbor, MI). MCF-7 cells were seeded in triplicate at a density of 40,000 cells per well in a 24-well plate and allowed to adhere overnight. Subsequently, the culture media were replaced with the appropriate treatments. Cells were treated with a mixture of 0.5% bacterial supernatant, trametinib at a concentration of 0.2 μM (Selleckchem) and Caspase 3/7 Green Dye (4440, Sartorius), diluted 1:1000 in serum-free DMEM. The Caspase-3/7 Green reagent incorporates an oligopeptide cleavage sequence (DEVD) linked to a DNA-binding dye, which, upon cleavage by caspase 3/7, labels apoptotic cells with a green fluorescence with an emission maximum at 530 nm. The plate was then placed in the Incucyte Live Cell Imager system, utilizing phase contrast and green fluorescence channels with a 10X objective. Nine images per well were captured every hour over a 24-hour period. Image analysis was conducted using the Incucyte® SX5 2022B software, employing the Cell-by-Cell Analysis Software Module (9600-0031) to mitigate background fluorescence. Parameters used were segmentation: “Top-Hat No Mask” and Radius (μm) “30. 000”. Quantification of green fluorescence served as an indicator of caspase 3/7 activation. The data were presented using GraphPad Prism 8.

### Image processing and statistical analysis

Protein quantification and gene expression studies were performed using Image Lab Software. The graphic representation and statistical analysis were carried out with GraphPad Prism software version 8. A two-tailed unpaired t-test, Mann-Whitney and two-way ANOVA test were used to calculate the *p*-values. (*) *p*-value < 0.05; (**) *p*-value < 0.01; (****) *p*-value < 0.0001; (n.s) *p*-value > 0.05.

## Supplementary information

Expended view figures (Combined)

Supplementary Figure legends

Original Data

## Data Availability

All relevant data are presented in the main manuscript or supplementary files; where data are not included, they will be made available to reviewers upon submission and to readers upon reasonable request.
