# Supplementary material for: HapA protease targets PAR-1/2 to modulate ERK signalling and reduce cancer cell viability
Source: Cell Death Discov. 2025 Aug 28;11:415. doi: 10.1038/s41420-025-02691-7 (PMC12394649; doi:10.1038/s41420-025-02691-7)

Begränsad delning

Supplementary Figure 1: Related to figure 1

A)

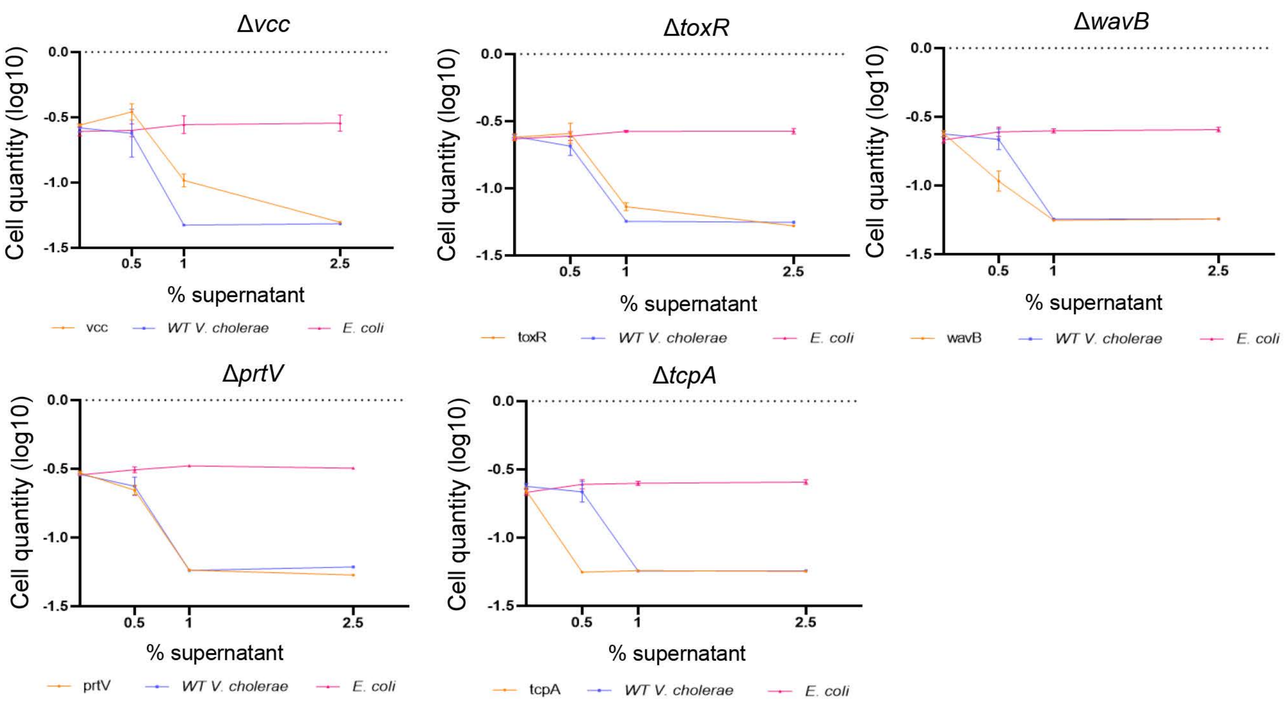

B)

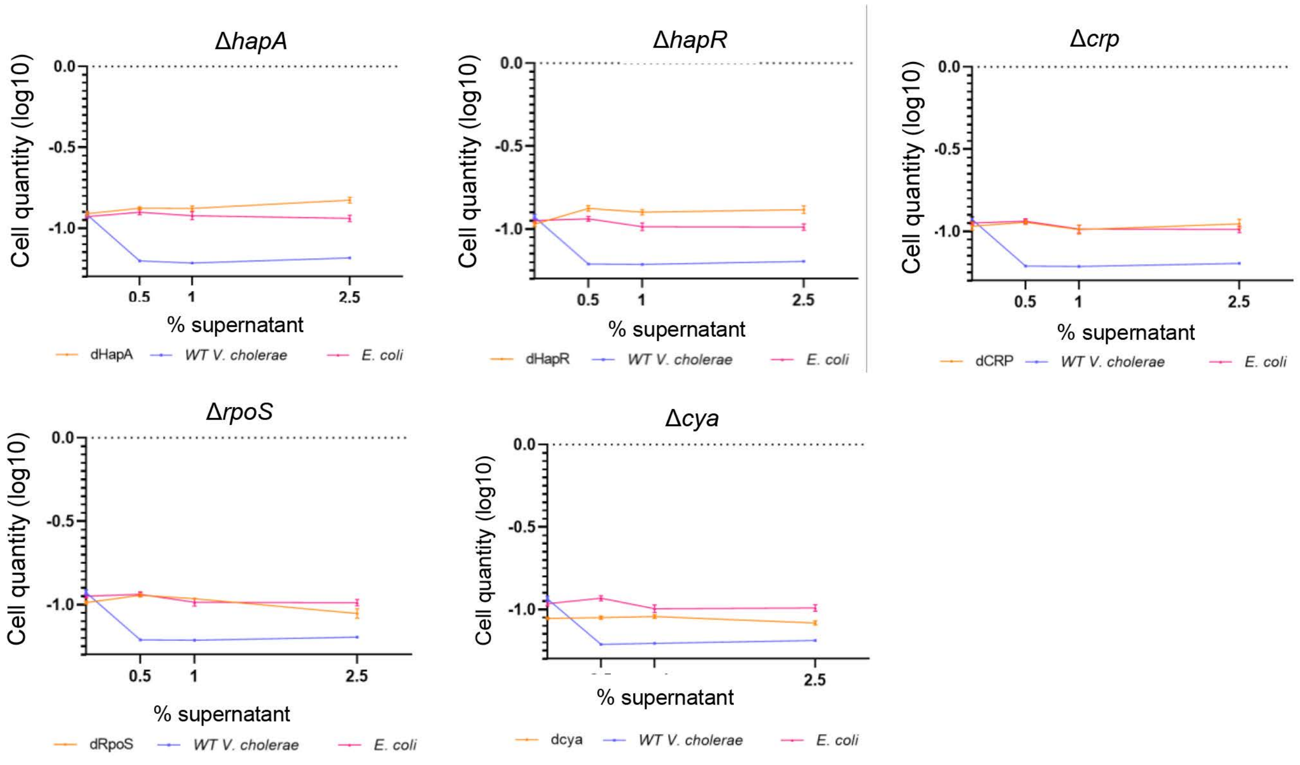

Supplementary Figure 2: Related to figure 1

A)

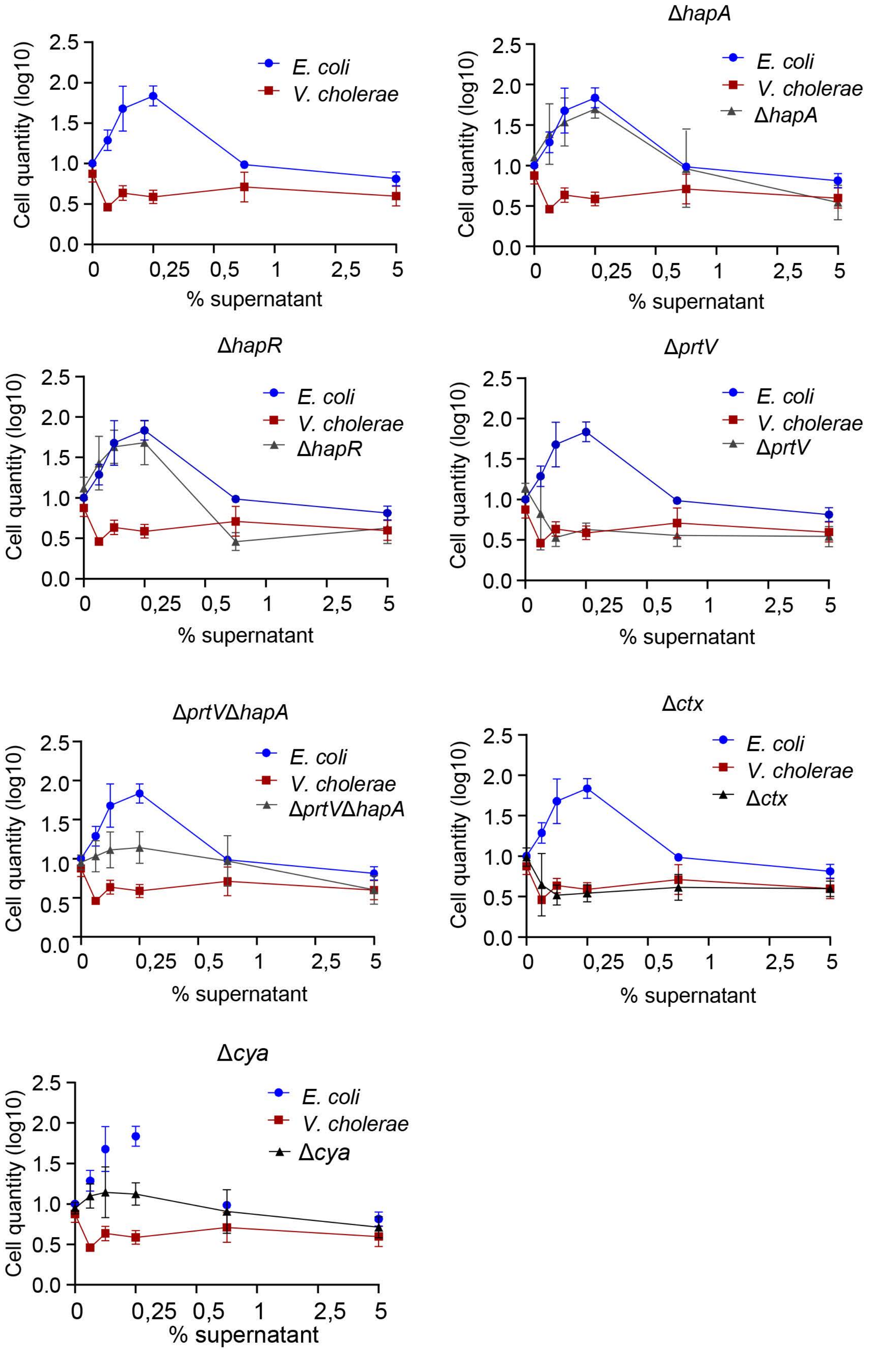

Begränsad delning

Continuation of Supplementary Figure 2: Related to figure 1

B)

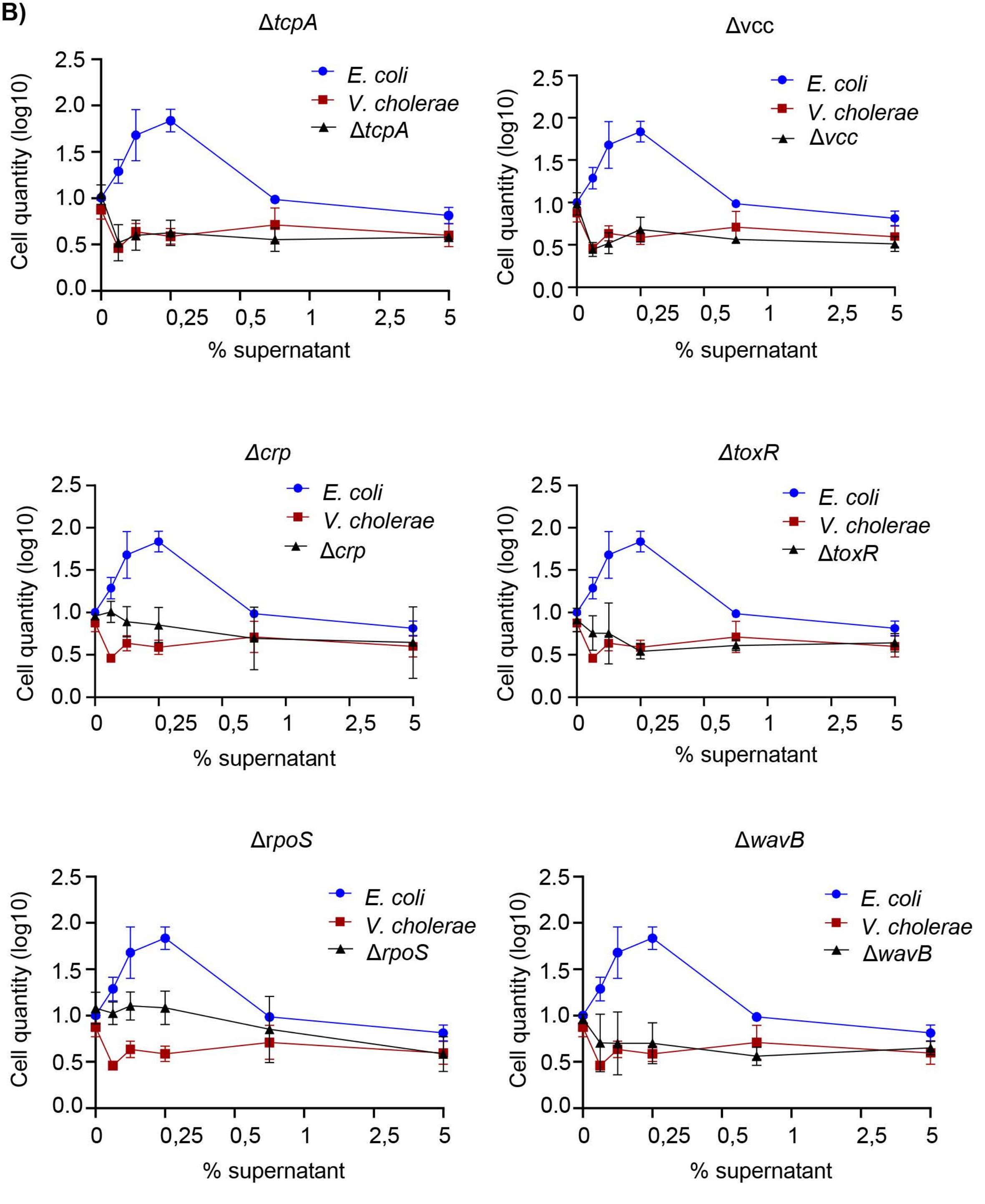

Supplementary Figure 3: Related to figure 1

A)

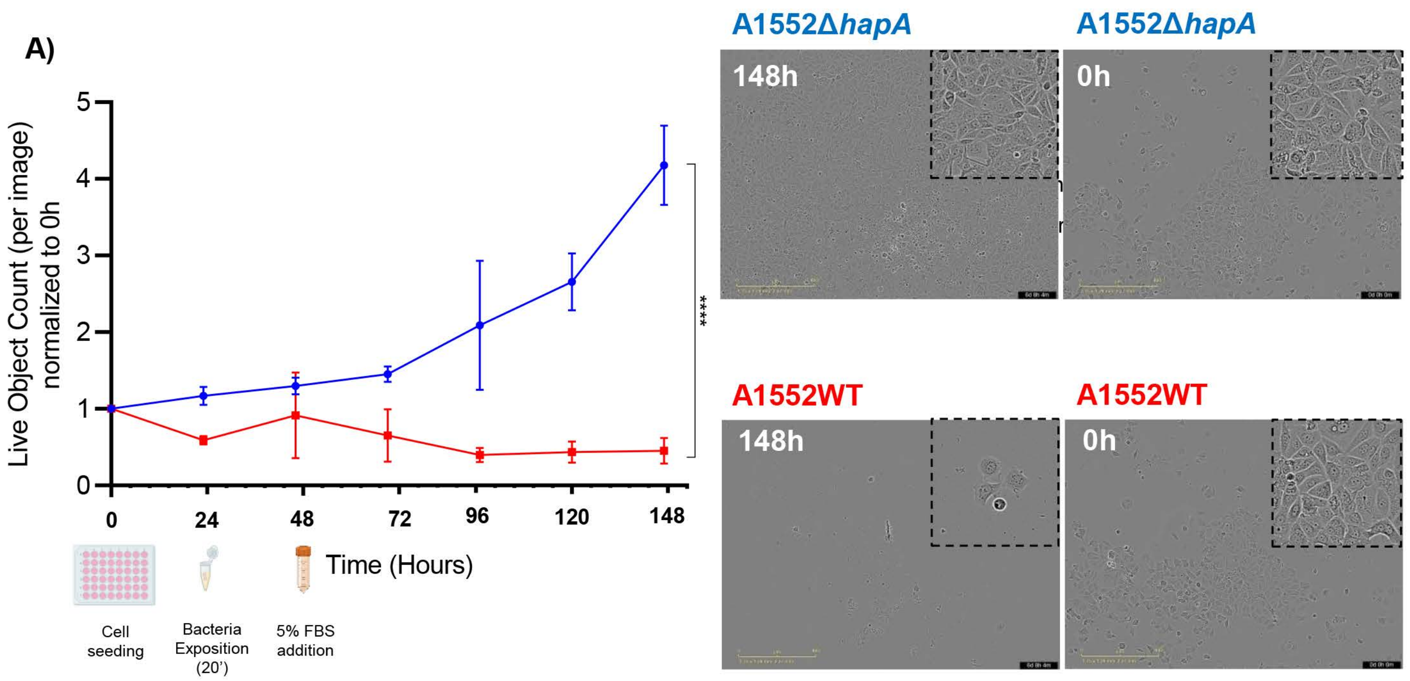

B)

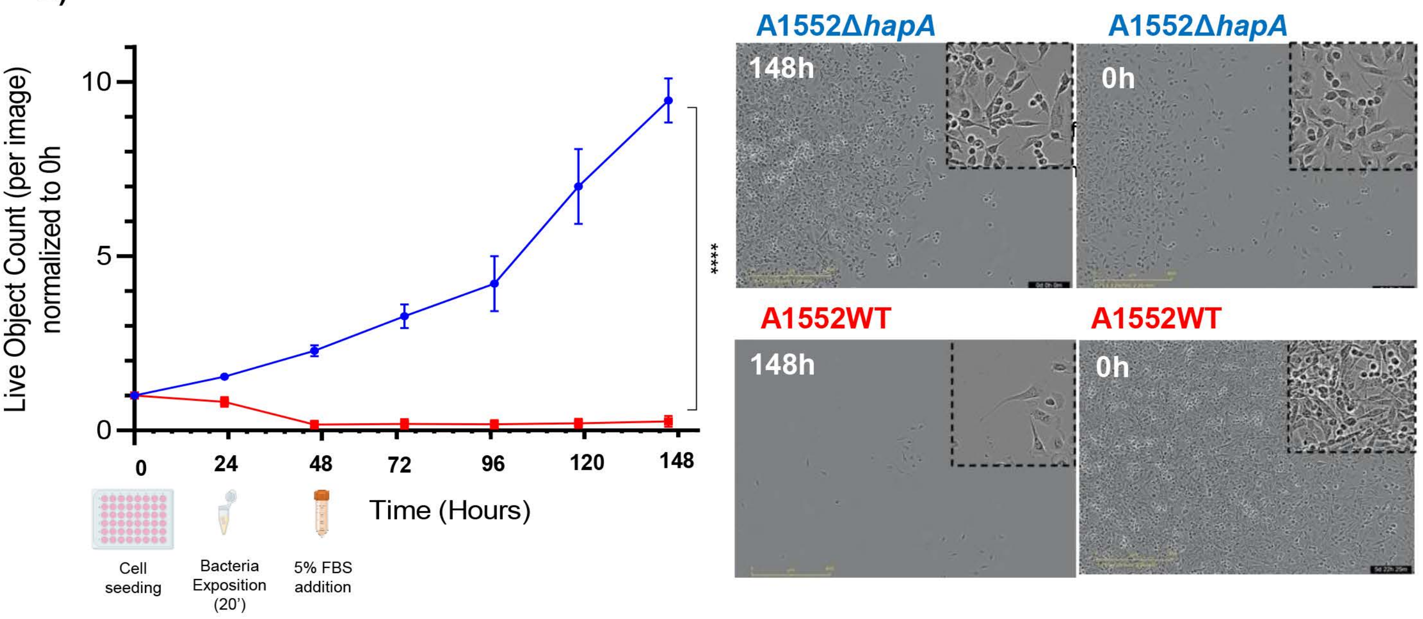

Begränsad delning

Continuation of Supplementary Figure 3: Related to figure 1

C)

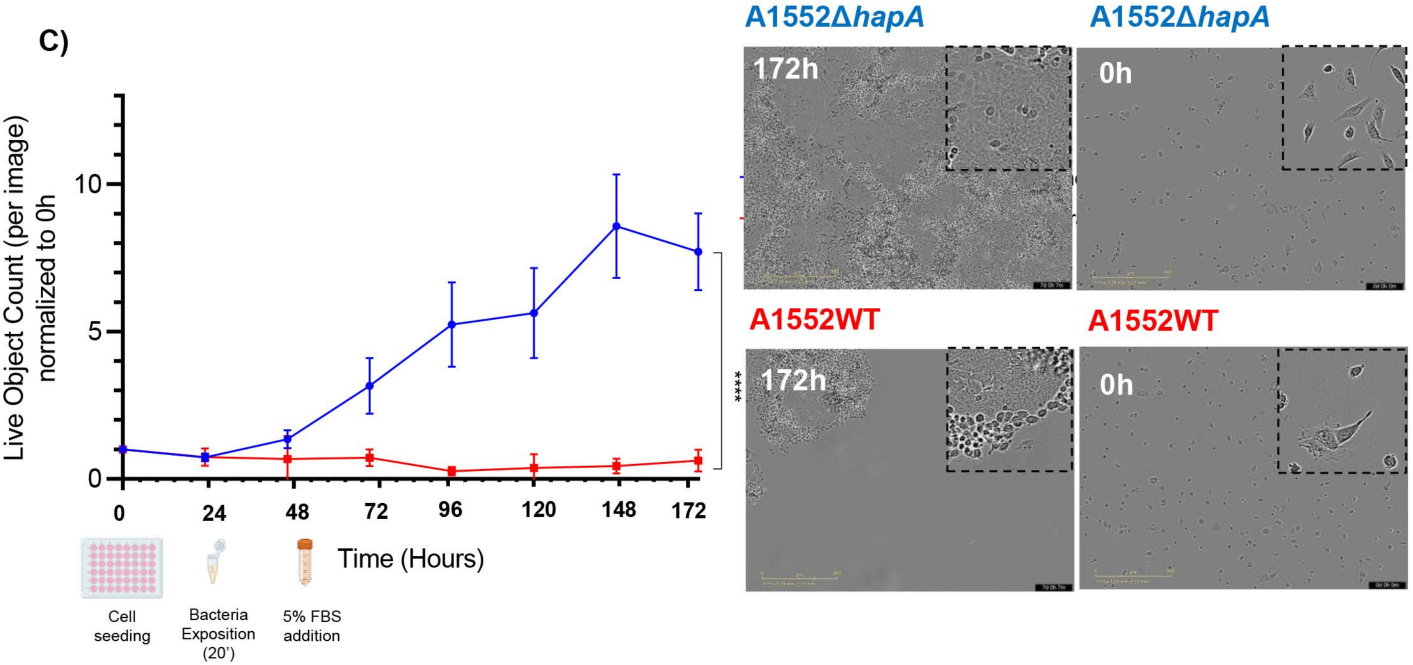

D)

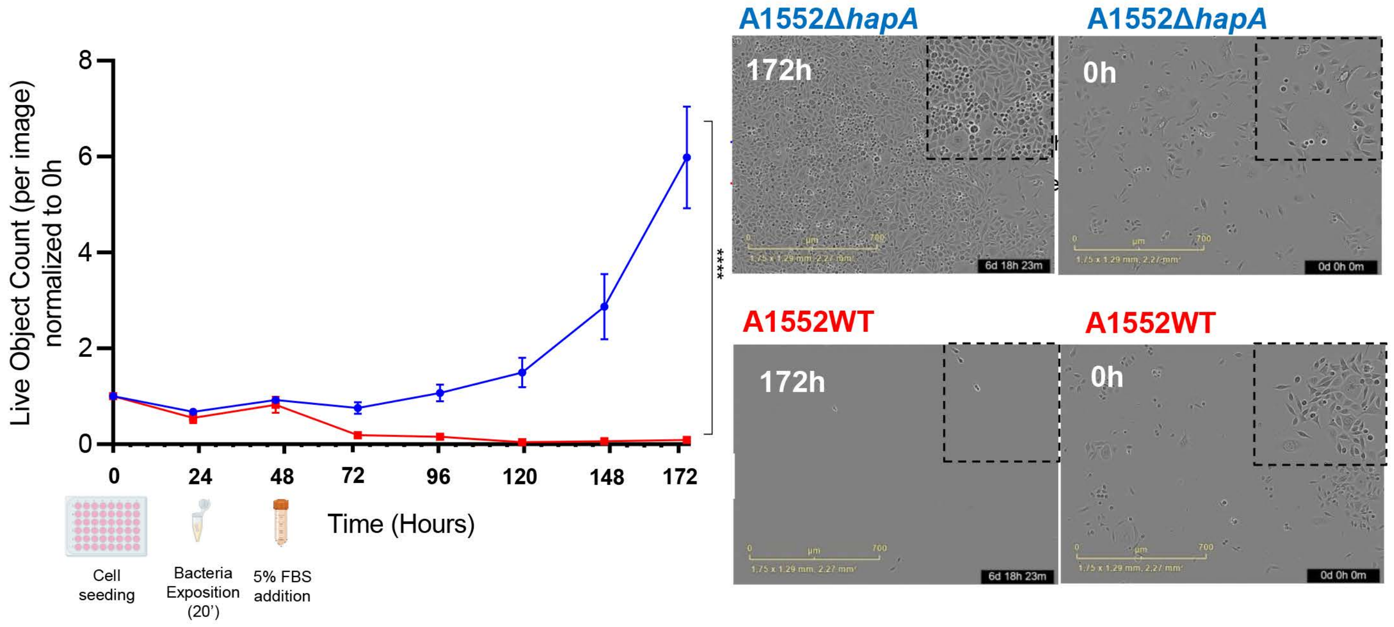

Supplementary Figure 4

A)

	Description	Scientific Name	Max Score	Total Score	Query Cover	E value	Per. Ident	Acc. Len	Accession
☑	Coagulation factor II (thrombin) receptor [Homo sapiens]	Homo sapiens	31.6	31.6	100%	22	100.00%	425	AAH02464.1
☑	thrombin receptor [Homo sapiens]	Homo sapiens	31.6	31.6	100%	22	100.00%	425	AAA36743.1
☑	proteinase-activated receptor 1 isoform 1 precursor [Homo sapiens]	Homo sapiens	31.6	31.6	100%	22	100.00%	425	NP_001983.2

B)

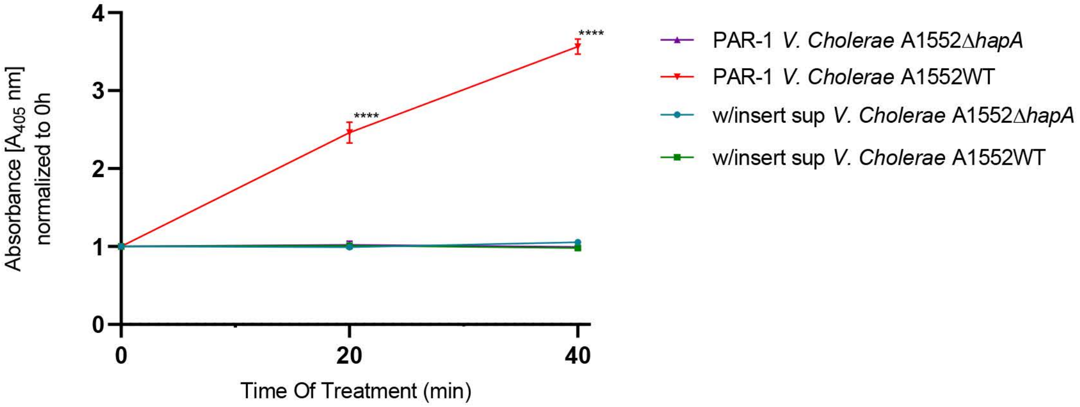

C)

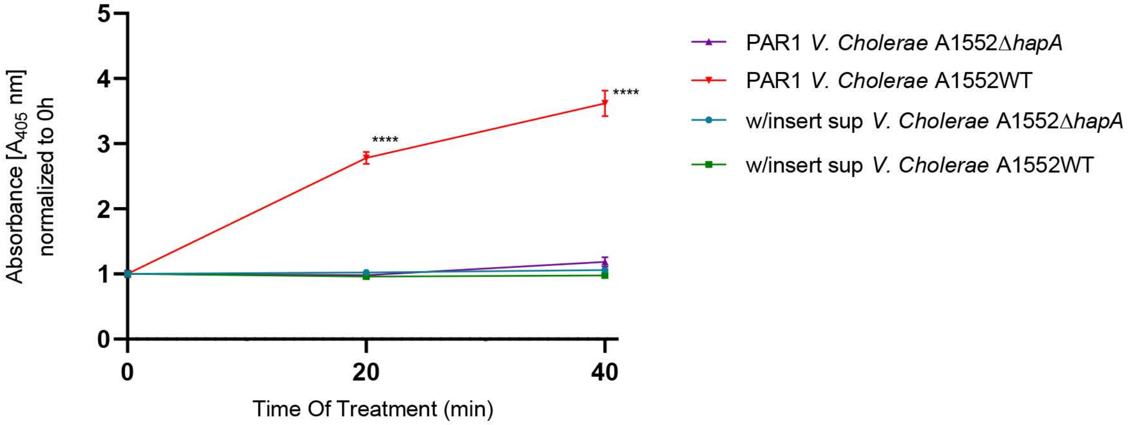

D)

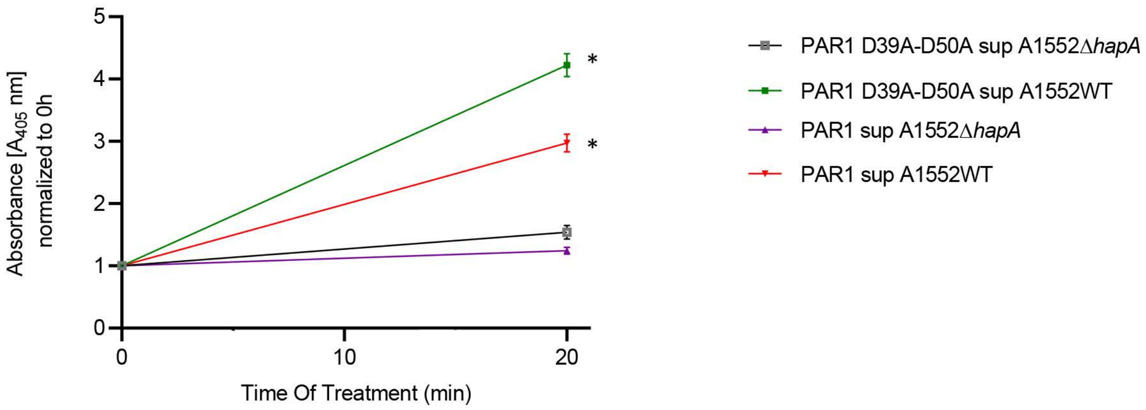

Supplementary Figure 5: Related to figure 2

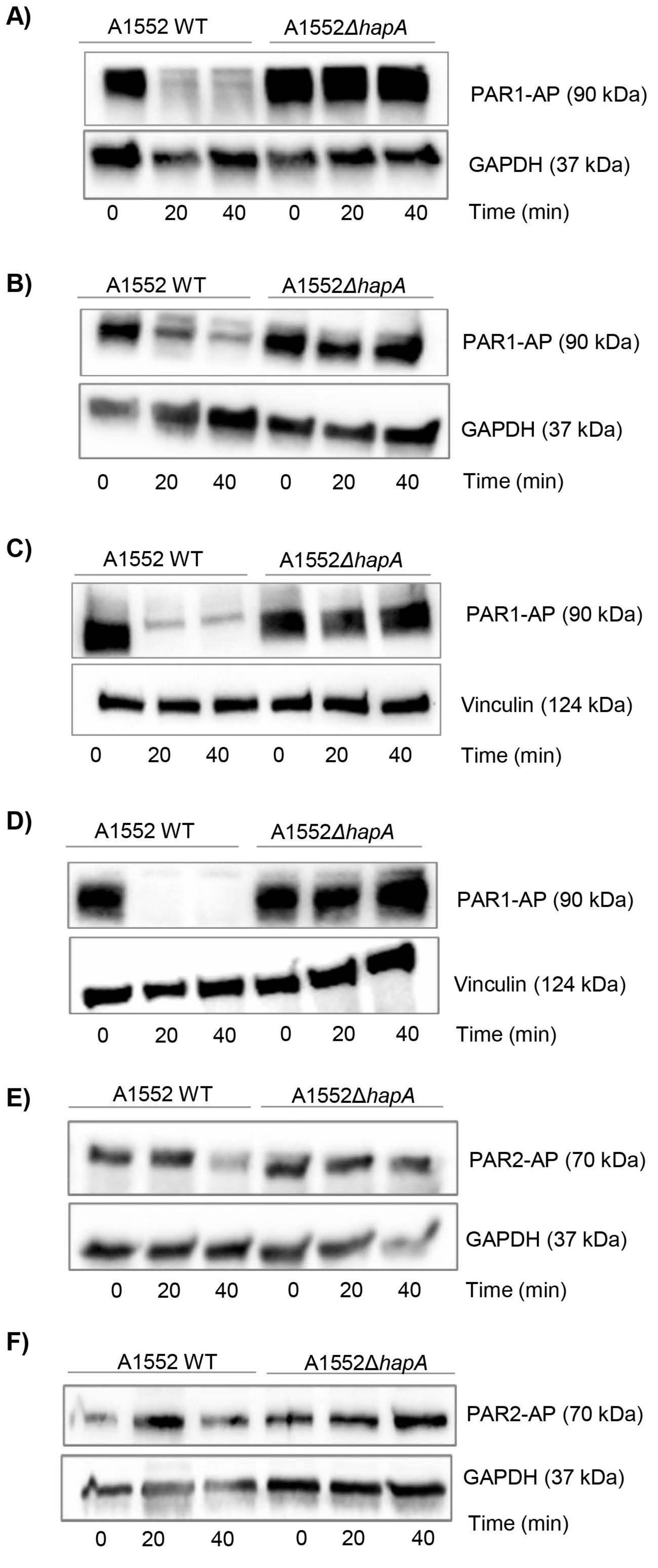

Supplementary Figure 6: Related to figure 3

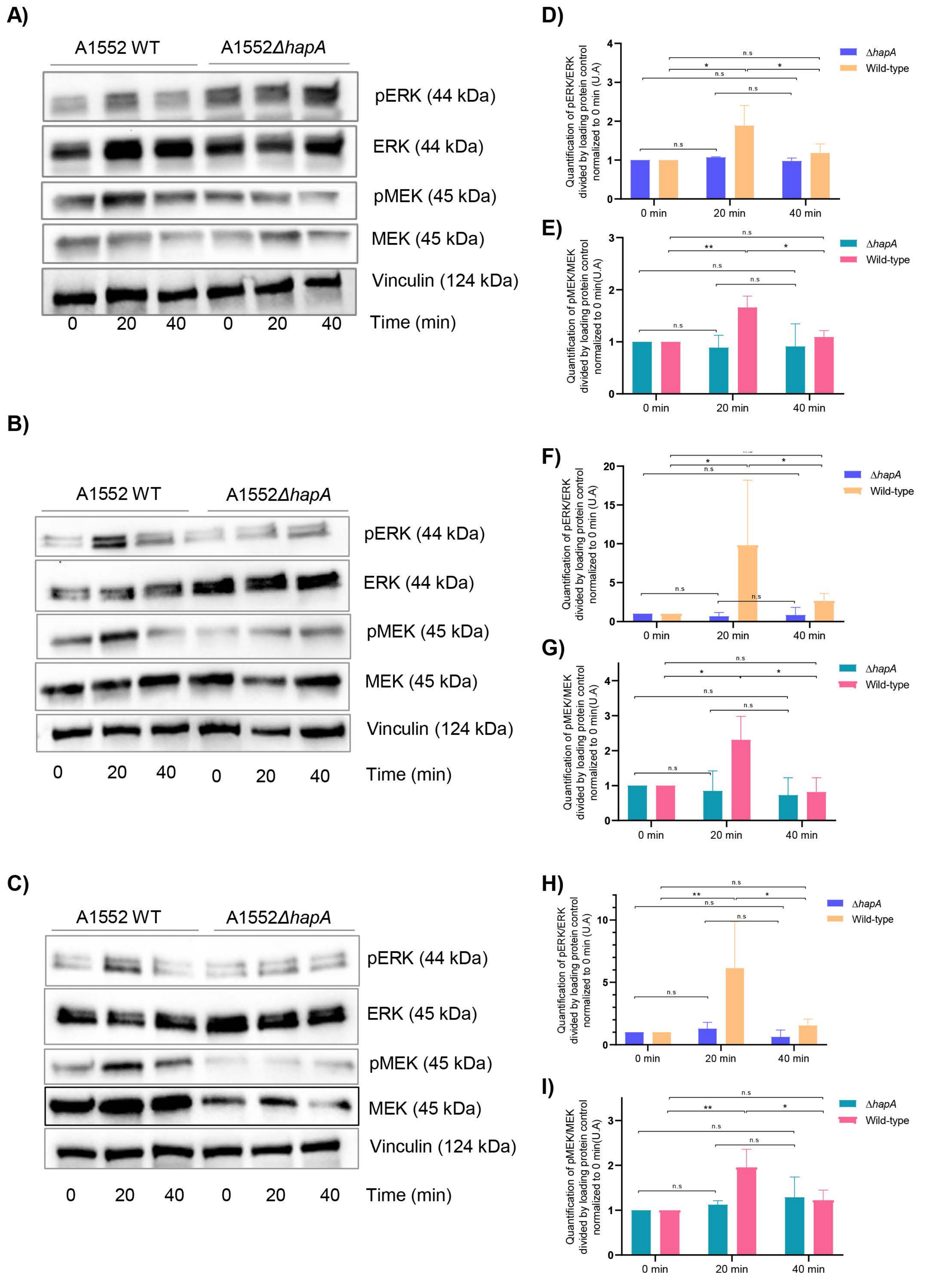

Supplementary Figure 7: Related to figure 3

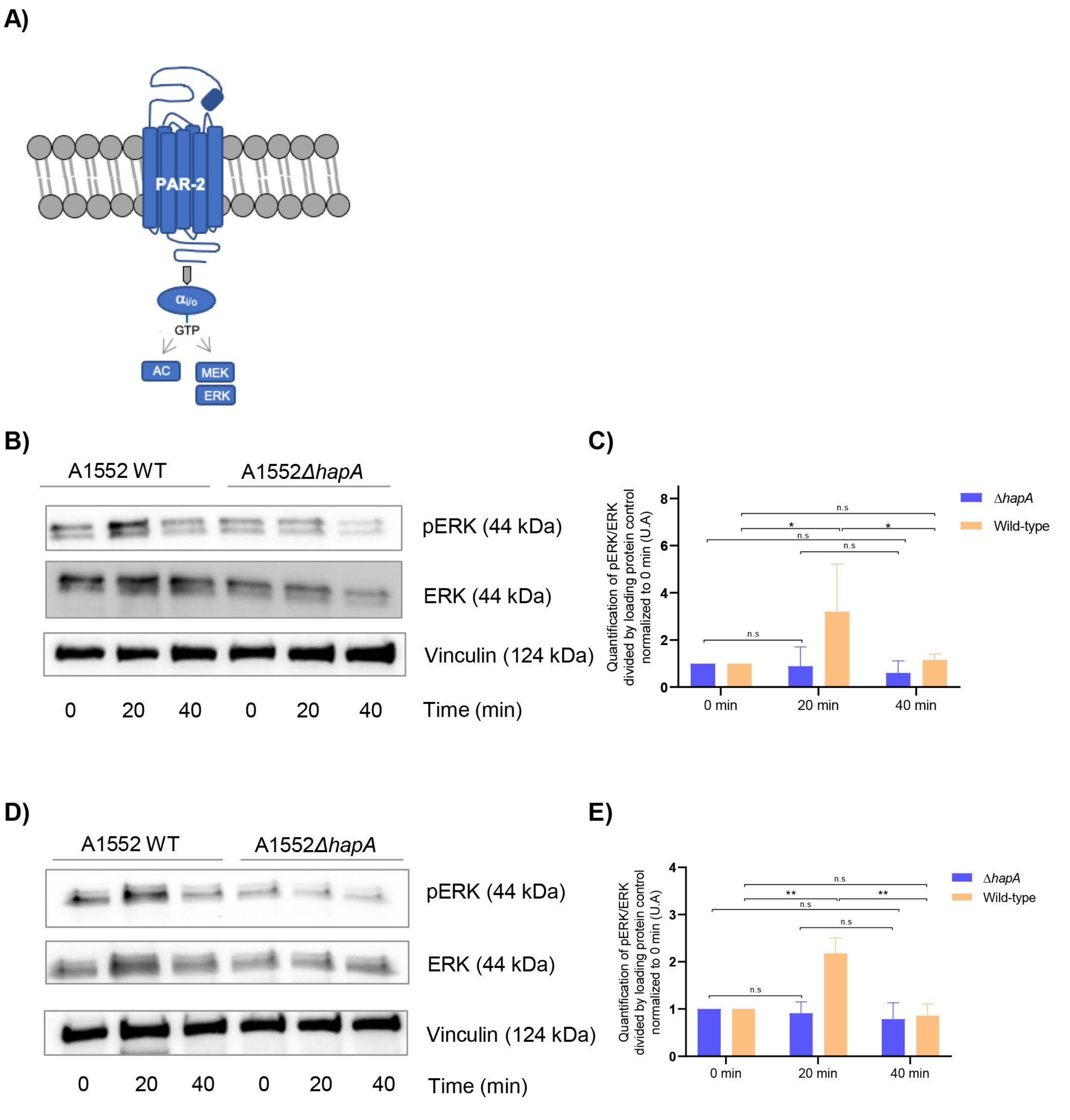

Begränsad delning

Supplementary Figure 8: Related to figure 5

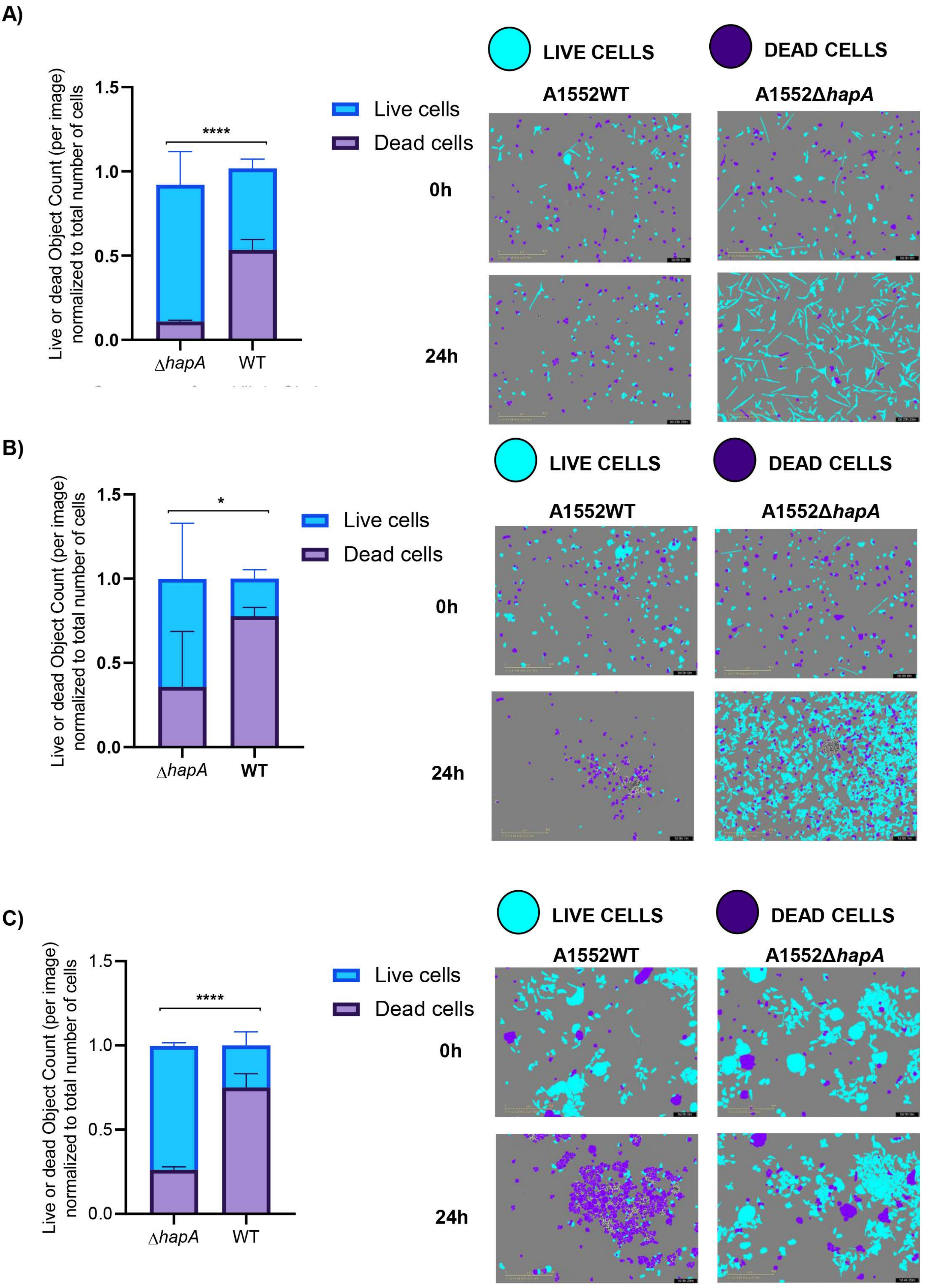

Supplementary Figure 8 continuation: Related to figure 5

D)

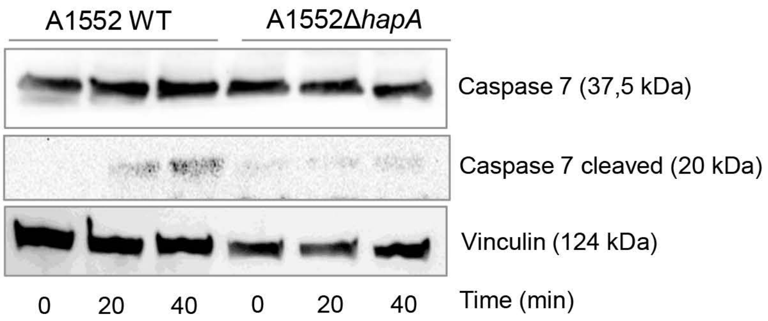

E)

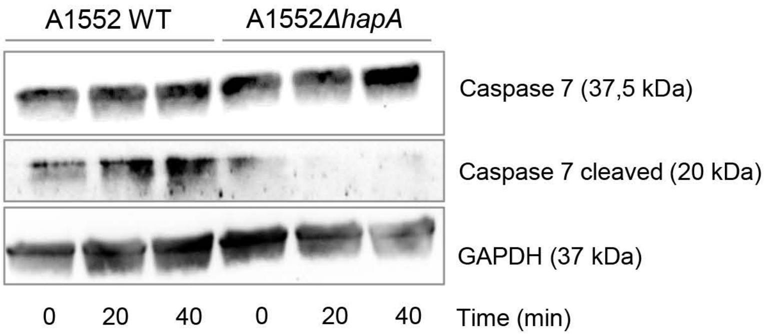

Supplementary Figure 9: Related to figure 5

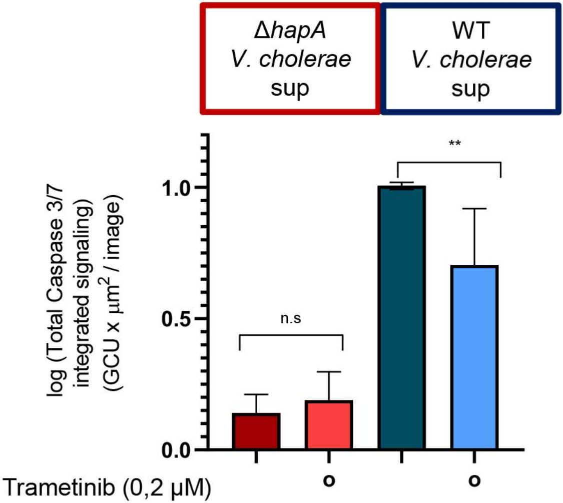

Supplement: Supplementary file 1 — Expended view figures (Combined) [file 41420_2025_2691_MOESM1_ESM.pdf]
