# Supplementary material for: HapA protease targets PAR-1/2 to modulate ERK signalling and reduce cancer cell viability
Source: Cell Death Discov. 2025 Aug 28;11:415. doi: 10.1038/s41420-025-02691-7 (PMC12394649; doi:10.1038/s41420-025-02691-7)
Supplement: Supplementary file 2 — Supplementary Figure legends [file 41420_2025_2691_MOESM2_ESM.pdf]

EXPANDED VIEW FIGURE LEGENDS

Expanded View Figure 1. Related to Figure 1. (**A**) Viability assay of negative hits in MCF-7 cells co-cultured with the supernatant from each of the non-positive *V. cholerae* mutant strains, the wild-type *V. cholerae* strain, or *E. coli* K12 strain at concentrations of 0, 0.5, 1 and 2.5% for 24 h. Live cells were fixed and stained using crystal violet, and cell quantity was measured at 590 nm. Experiments were performed in triplicate; results were normalized using Log10, and data are presented as mean ± SEM. (*) p-value < 0.05; (**) p-value < 0.01; (***) p-values < 0.001; (ns) p-value > 0.05. (**B**) Viability assay of positive hits identified at MCF-7 cells tested in MDA-MB-231 cells co-cultured with the supernatant from each of the 14 *V. cholerae* mutant strains, the wild-type *V. cholerae*, or the *E. coli* K12 strain at concentrations of 0, 0.5, 1 and 2.5% for 24 h. Live cells were fixed and stained using crystal violet, and cell quantity was measured at 590 nm. Experiments were performed in triplicate; results were normalized using Log10, and data are presented as mean ± SEM. (*) p-value < 0.05; (**) p-value < 0.01; (***) p-value < 0.001; (n.s) p-value > 0.05.

Expanded View Figure 2. Related to Figure 1. Viability assay of (**A**) positive and (**B**) negative hits in HCT-8 cells co-cultured with the supernatant from each of the 14 *V. cholerae* mutant strains, the wild-type *V. cholerae* strain, or the *E. coli* K12 strain at concentrations of 0, 0.5, 1 and 2.5% for 24 h. Live cells were fixed and stained using crystal violet, and cell quantity was measured at 590 nm. Experiments were performed in triplicate; results were normalized applying Log10 and data are presented as mean ± SEM. (*) p-value < 0.05; (**) p-value < 0.01; (***) p-value < 0.001; (ns) p-value > 0.05.

Expanded View Figure 3. Proliferation assay over 7 days of (**A**) MCF-7, (**B**) MDA-MB-231, (**C**) HCT-8, or (**D**) SUIT-2 cells co-cultured with 0.5% bacterial supernatant from either wild-type *V. cholerae* (WT VC) or the Δ*hapA* mutant strain for 20 minutes. After 24 hours, cells were transferred to medium containing 5% FBS. On the following day, a second exposure to the supernatant was performed. Live cell counts (per image) were monitored using the Incucyte® Live-Cell Analysis System for approximately 148–172 hours. Experiments were performed in triplicate. Data were presented as mean ± SEM. Statistical significance: (****) p-value < 0.0001.

Expanded View Figure 4. (A) Identification of human proteins with HapA cleaved site (protein blast). PAR-1 cleavage assay in (**B**) HCT-8 cells and (**C**) SUIT-2 cells. (****) p-value < 0.0001. (**D**) PAR-1 cleavage assay in MCF-7 cells transfected with the PAR1 reporter construct carrying the mutations for MMP-1 cleavage (D39A-D50A) or wild-type plasmid DNA were treated with the supernatant from the wild-type *V. cholerae* or from Δ*hapA* mutant strain at a concentration of 0.5% for 20 min. Alkaline phosphatase activity following PAR cleavage was

quantified by incubation with the colorimetric substrate 1-Step PNPP and measured at 405 nm. The experiments were performed in triplicates; results were normalized applying Log10 and data are presented as mean ± SEM. (*) p-value < 0.05 (comparison double mutant vs wild-type PAR-1).

Expanded View Figure 5. Related to Figure 2. (**A-D**) PAR-1 and PAR-2 protein levels in transfected cells. MCF-7 cells (**A**), MDA-MB-231 cells (**B**), HCT-8 cells (**C**) and SUIT-2 (**D**) cells were transfected with the PAR-1 reporter construct and treated with the supernatant from the the wild-type *V. cholerae* (WT VC), or from the Δ*hap*A mutant strain at a concentration of 0.5% for 0, 20, and 40 minutes. Cells were collected, and protein extracts were analysed by SDS-PAGE and western blot for the expression of PAR-1. GAPDH or vinculin was used as a loading control. (**E-F**) MCF-7 cells (**E**) and MDA-MB-231 cells (**F**) cells were transfected with the PAR-2 reporter construct and treated with the supernatant from the the wild-type *V. cholerae*, or from the Δ*hap*A mutant strain at a concentration of 0.5% for 0, 20, and 40 minutes. Cells were collected, and protein extracts were analysed by SDS-PAGE and western blot for the expression of PAR2. GAPDH was used as a loading control. All experiments were performed in triplicates.

Expanded View Figure 6. Related to Figure 2. MDA-MB-231 cells (**A**), HCT-8 cells (**B**) and SUIT-2 cells (**C**) were transfected with the PAR-1 reporter construct and were treated with the supernatant from the wild-type *V. cholerae* (A1552 WT) or from the Δ*hapA* mutant (A1552 Δ*hapA*) strain at a concentration of 0.5% for 0, 20 and 40 minutes. Cells were collected, and protein extracts were analysed by SDS-PAGE and western blot for the expression of ERK, p-ERK, MEK and p-MEK. Vinculin was used as a loading control. The figure provides quantitative analysis of the experiment in MDA-MB-231 cells (**D, E**), HCT-8 cells (**F, G**) and SUIT-2 cells (**H, I**) cells with visualized bands for ERK, p-ERK (**D, F, H**), MEK and p-MEK of (**E, G, I**). Vinculin was used as a loading control. The experiments were performed in triplicates. A representative experiment is shown. (*) p-value < 0.05; (**) p-value < 0.01; (n.s) p-value > 0.05.

Expanded View Figure 7. Related to Figure 2. PAR-2 protein levels in transfected cells. (**A**) Schematic illustration of PAR-2 signalling activating MEK/ERK signalling. (**B-E**) MCF-7 cells (**B**) and MDA-MB-231 cells (**D**) were transfected with the PAR-2 reporter construct and were treated with the supernatant from the wild-type *V. cholerae* (A1552 WT) or from the Δ*hapA* mutant (A1552 Δ*hapA*) strain at a concentration of 0.5% for 0, 20 and 40 minutes. Cells were collected, and protein extracts were analysed by SDS-PAGE and western blot for the expression of ERK and p-ERK. Vinculin was used as a loading control. The right panel of the figure provides quantitative analyses of the experiment in MCF-7 cells with visualized bands for ERK, p-ERK in PAR-2 MCF-7 cells (**C**) and MDA-MB-231 cells (**E**). The experiments were

performed in triplicates. A representative experiment is shown whereby (*) p-value < 0.05; (**) p-value < 0.01; (n.s) p-value > 0.05.

Expanded View Figure 8. Related to Figure 5. (**A-C**) Quantification of live and dead (**A**) MDA-MB-231, (**B**) HCT-8, (**C**) SUIT-2 cells percentages using the Incucyte® Artificial Intelligence Cell Health Analysis Module with 10X objective, 24 hours after a 20-minute incubation with 0.5% supernatant from either wild-type *V. cholerae* or the Δ*hapA* mutant strain. (**D-E**) Measurement of Caspase 7 cleavage by western blot in MCF-7 cells (**D**) and MDA-MB-231 cells (**E**). Cells treated with supernatant from wild-type *V. cholerae* or HapA mutant strain at a concentration of 0,5% for 0-, 20- and 40-min. Western Blot was used to identify levels of total and cleaved Caspase 7. GAPDH was used as a loading control. The experiments were performed in triplicates. A representative experiment is shown.

Expanded View Figure 9. Related to Figure 5. HapA induces caspase 3/7 activity in MCF cells.

Graph that represents total integrated Caspase 3/7 activity at 11h measured by emission of fluorescence at 530 nm from**.** The experiments were performed in triplicates. A representative experiment is shown. Results were normalized, and data are presented as mean ± SEM. (**) p-value < 0.01; (n.s) p-value > 0.05.
