# Supplementary material for: HapA protease targets PAR-1/2 to modulate ERK signalling and reduce cancer cell viability
Source: Cell Death Discov. 2025 Aug 28;11:415. doi: 10.1038/s41420-025-02691-7 (PMC12394649; doi:10.1038/s41420-025-02691-7)

Begränsad delning

Uncropped membrane for Figure 1D

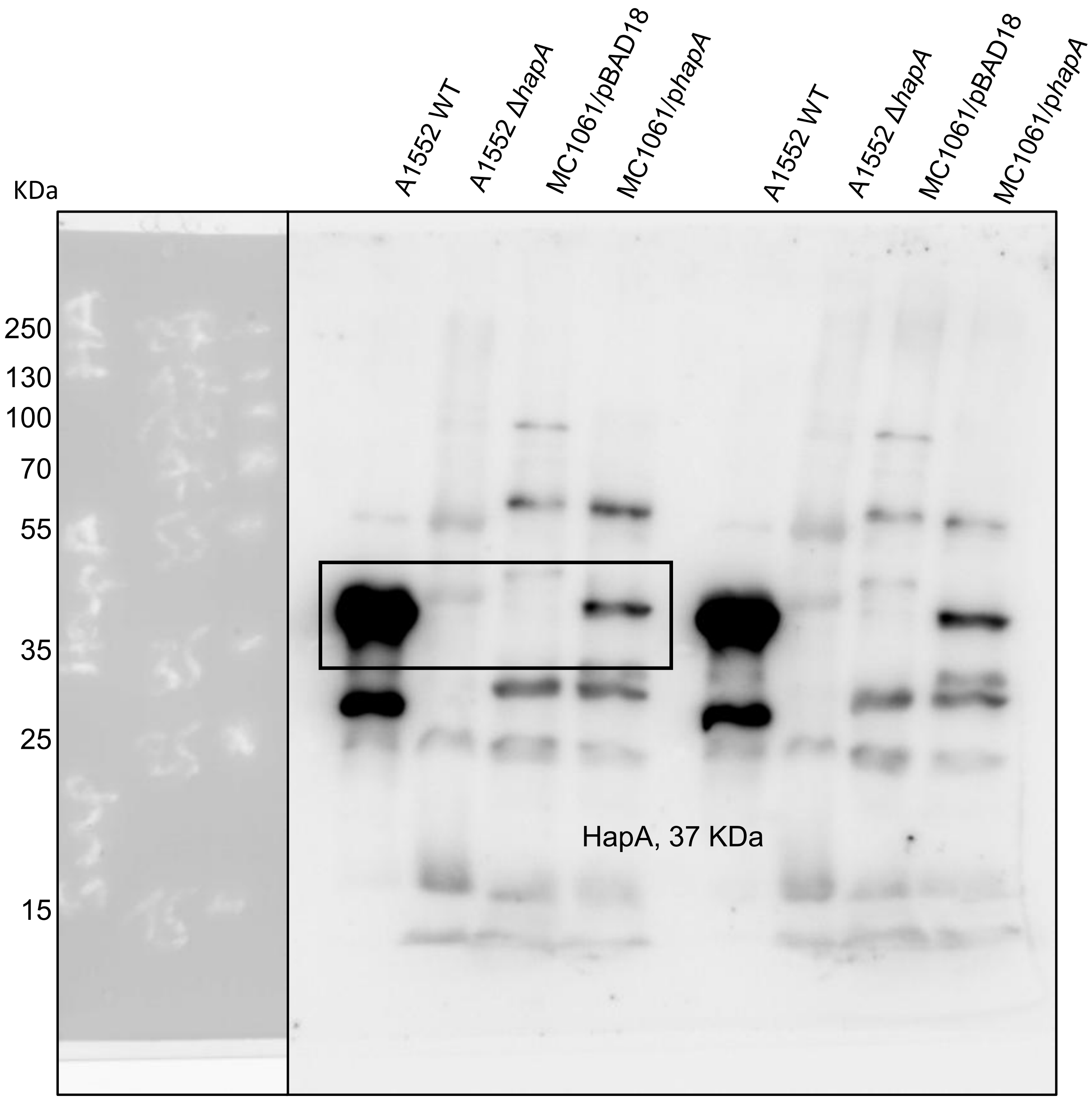

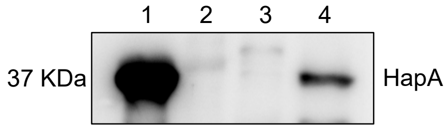

1) A1552 WT

2) A1552Δ*hapA*

3) MC1061/pBAD18

4) MC1061/p*hapA*

Begränsad delning

Uncropped membrane for Figure 1F

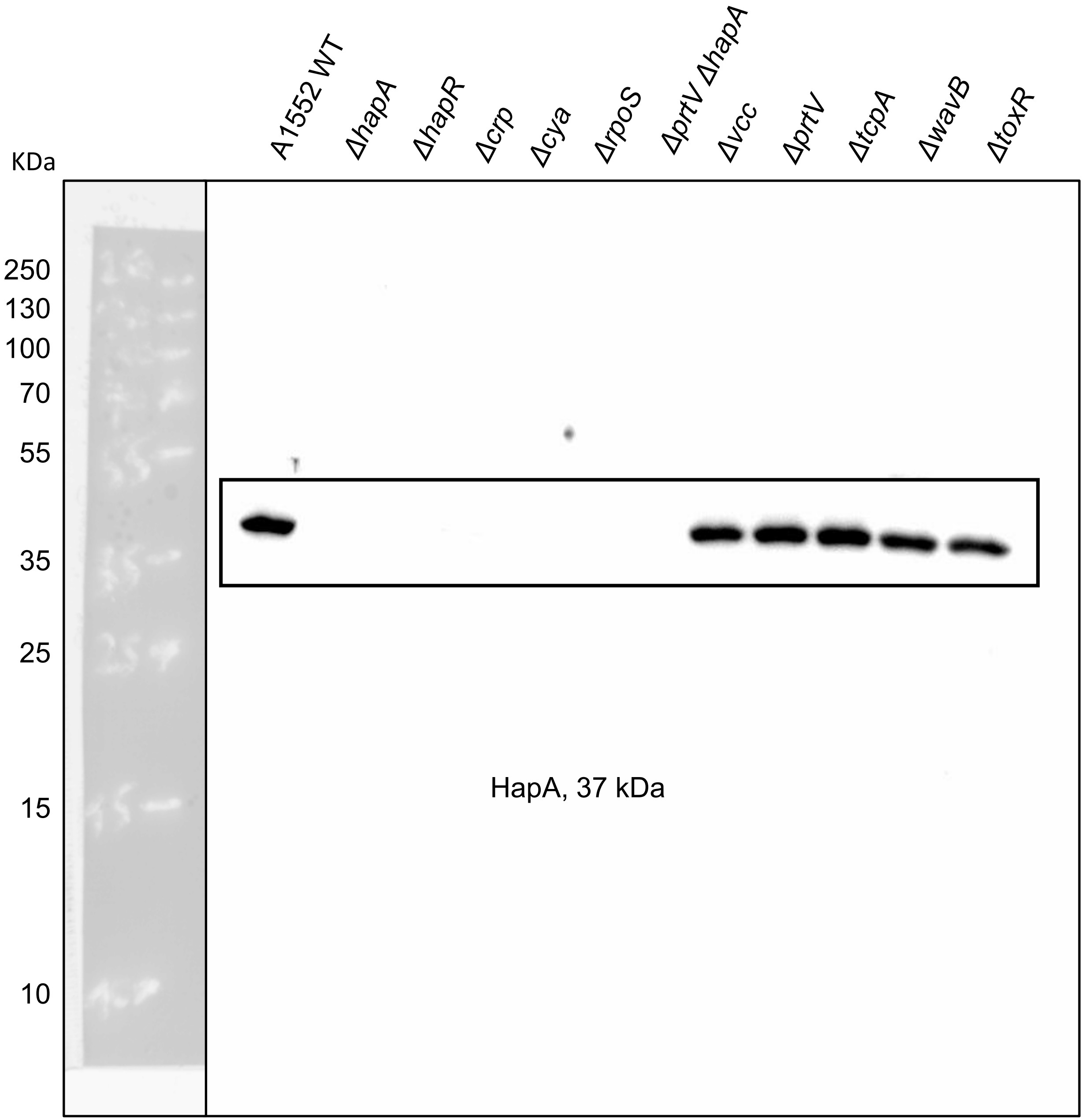

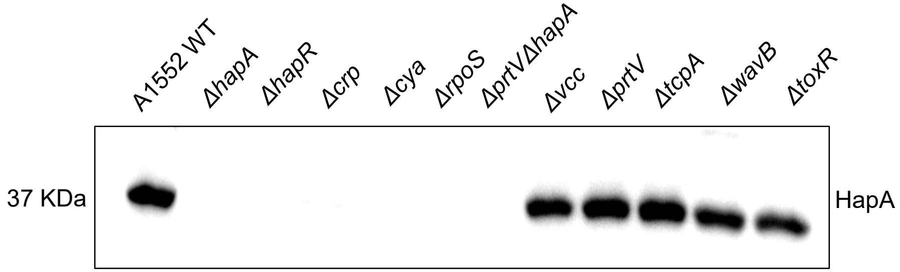

Uncropped membranes for Figure 3B. Membranes were cut into two parts prior to detection.

Membrane 1

Piece 1

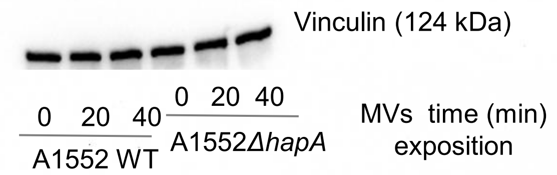

Piece 2

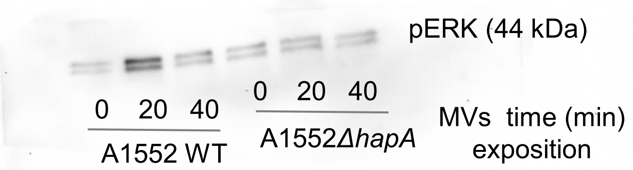

Piece 2

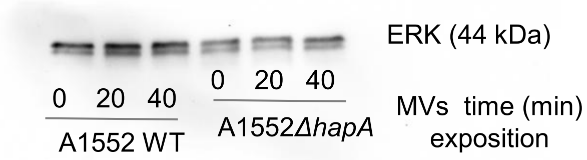

Membrane 2

Piece 1

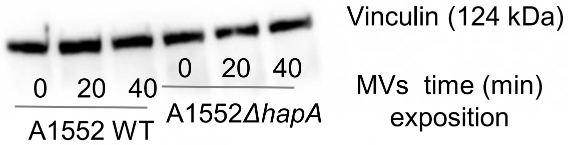

Piece 2

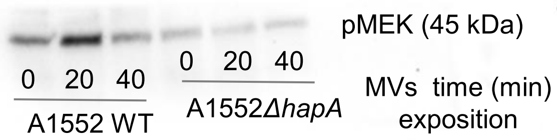

Piece 2

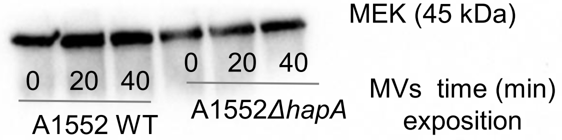

Begränsad delning

Uncropped membranes for Figure 4A. Membranes were cut into two parts prior to detection.

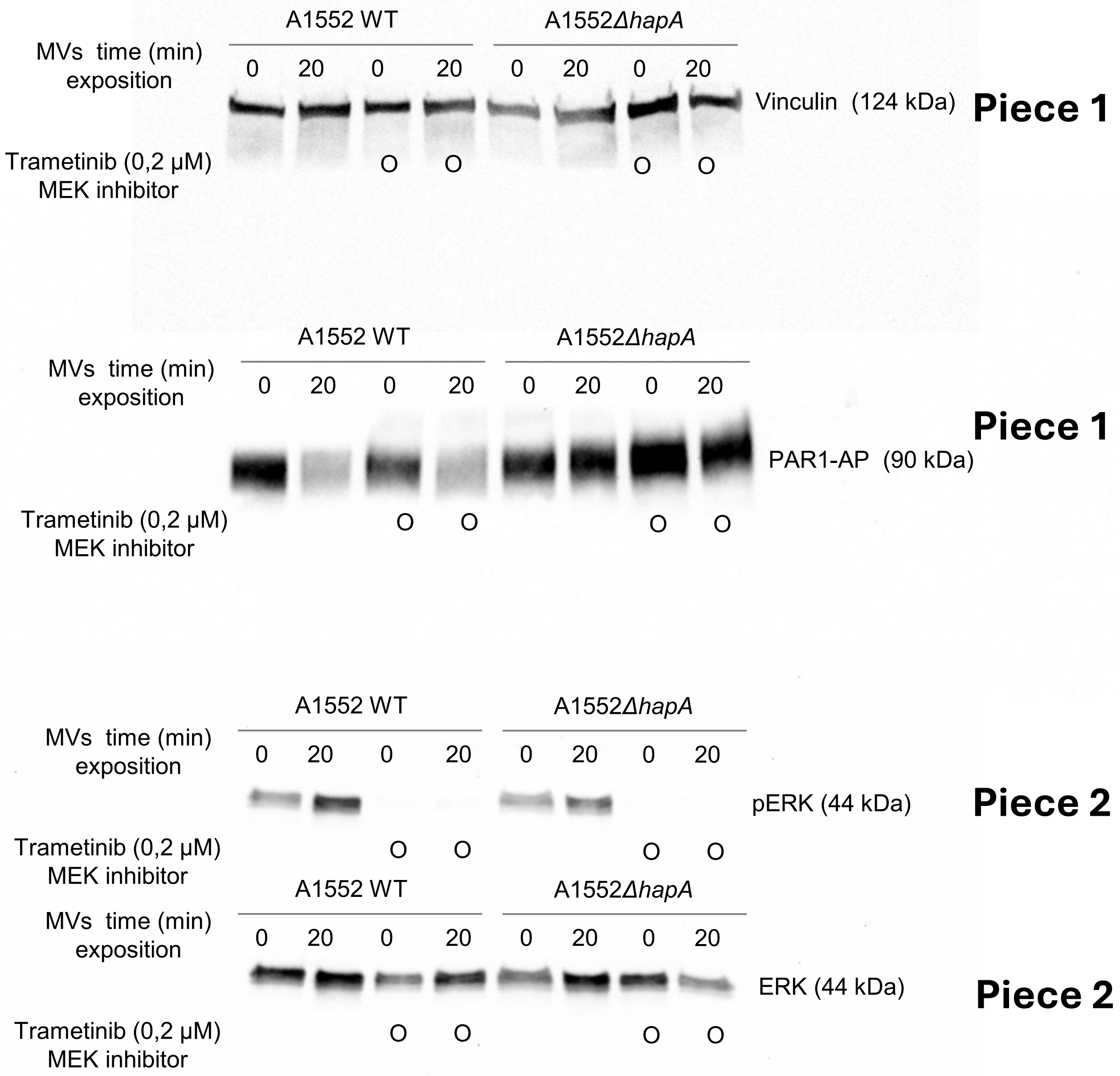

Begränsad delning

Uncropped membranes for Supplementary figure 5 A and B . Membranes were cut into two parts prior to detection.

A) Membrane cut into two pieces

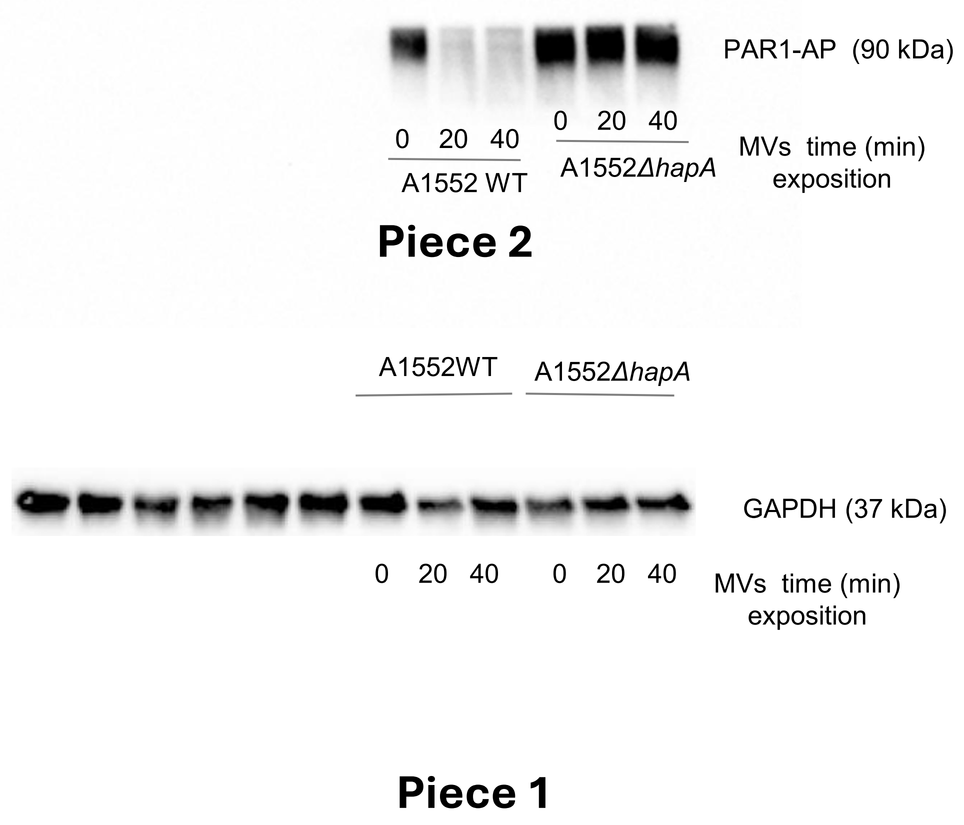

B) Membrane cut into two pieces

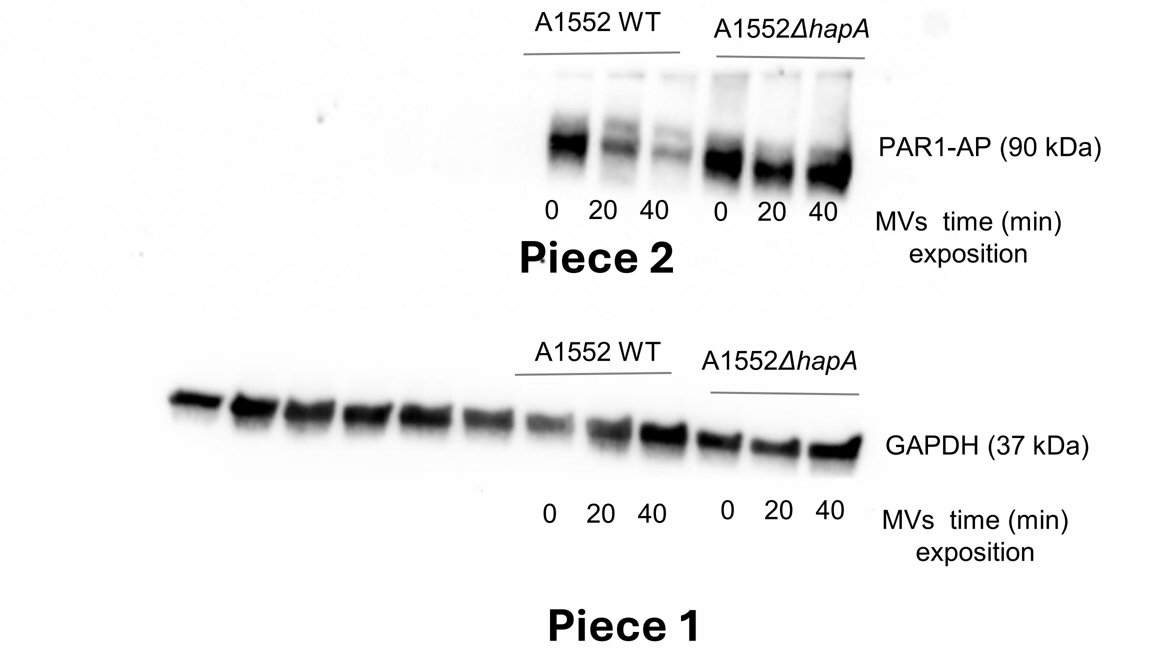

Begränsad delning

Uncropped membranes for Supplementary figure 5 C and D . Membranes were cut into two parts prior to detection.

C) Membrane cut into two pieces

Piece 2

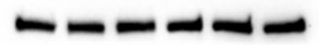

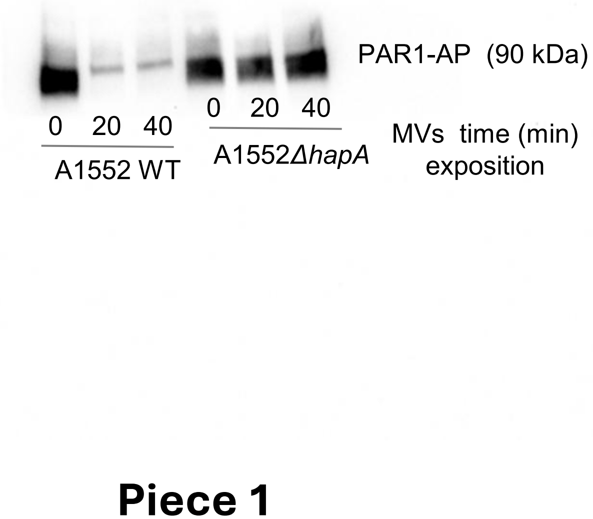

Piece 1

D) Membrane cut into two pieces

Piece 2

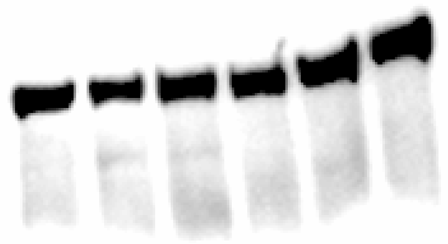

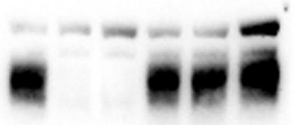

Piece 1

Begränsad delning

Uncropped membranes for Supplementary figure 5 E and F . Membranes were cut into two parts prior to detection.

E) Membrane cut into two pieces

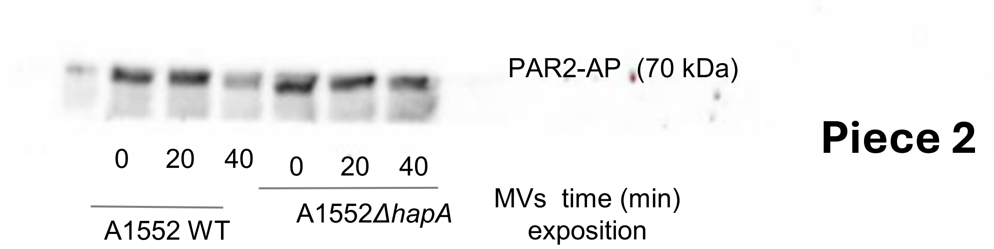

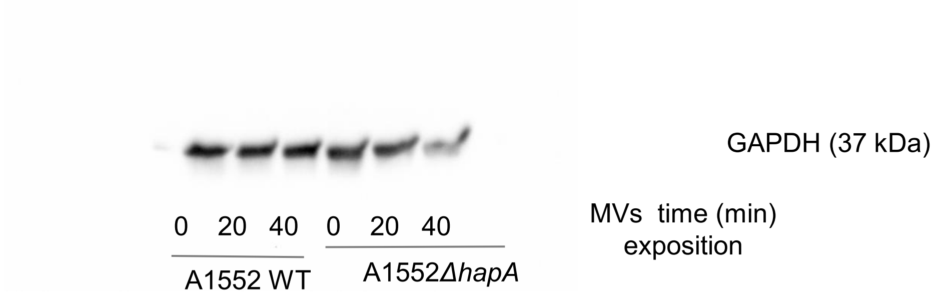

F) Membrane cut into two pieces

Piece 2

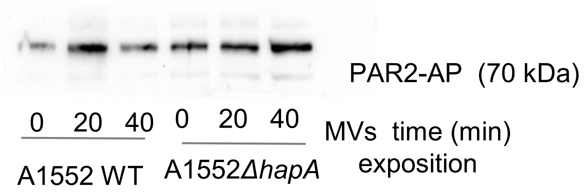

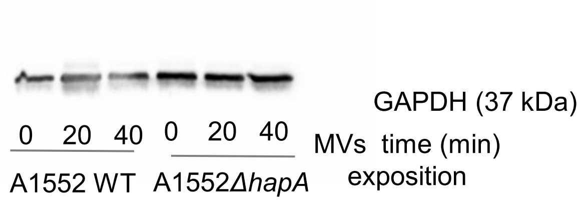

Piece 1

Begränsad delning

Uncropped membranes for Supplementary figure 6A. Membranes were cut into two parts prior to detection.

Membrane 1

Piece 2

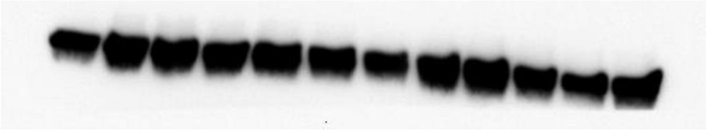

GAPDH (37 kDa)

0 20 40 | 0 20 40 — MVs time (min) exposition
A1552 WT | A1552Δ*hapA*

Piece 1

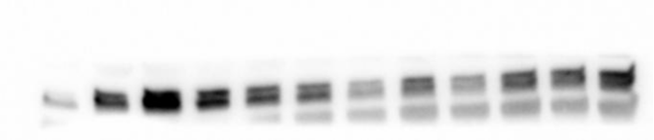

pERK (44 kDa)

0 20 40 | 0 20 40 — MVs time (min) exposition
A1552 WT | A1552Δ*hapA*

Piece 1

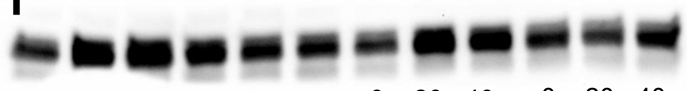

ERK (44 kDa)

0 20 40 | 0 20 40 — MVs time (min) exposition
A1552 WT | A1552Δ*hapA*

Membrane 2

Piece 2

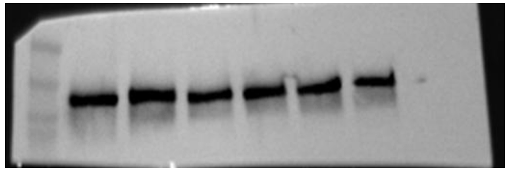

Vinculin (124 kDa)

0 20 40 | 0 20 40 — MVs time (min) exposition
A1552 WT | A1552Δ*hapA*

Piece 1

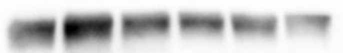

pMEK (45 kDa)

0 20 40 | 0 20 40 — MVs time (min) exposition
A1552 WT | A1552Δ*hapA*

Piece 1

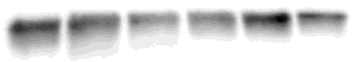

MEK (45 kDa)

0 20 40 | 0 20 40 — MVs time (min) exposition
A1552 WT | A1552Δ*hapA*

Begränsad delning

Uncropped membranes for Supplementary figure 6B. Membranes were cut into two parts prior to detection.

Membrane 1

Piece 1

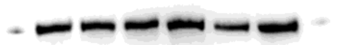

Piece 2

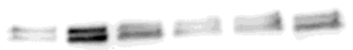

Piece 2

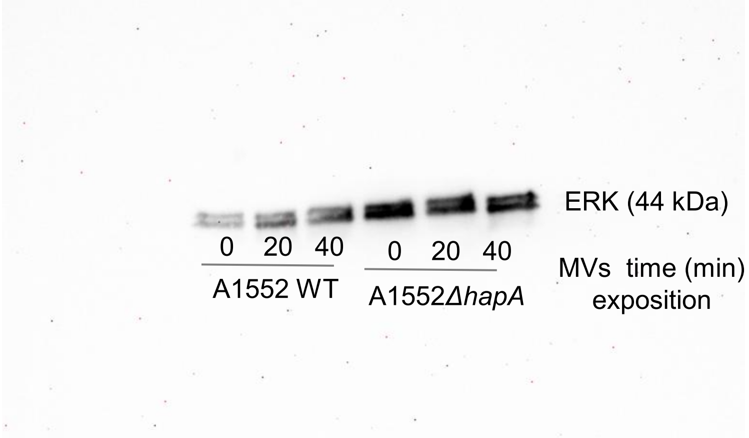

Membrane 2

Piece 2

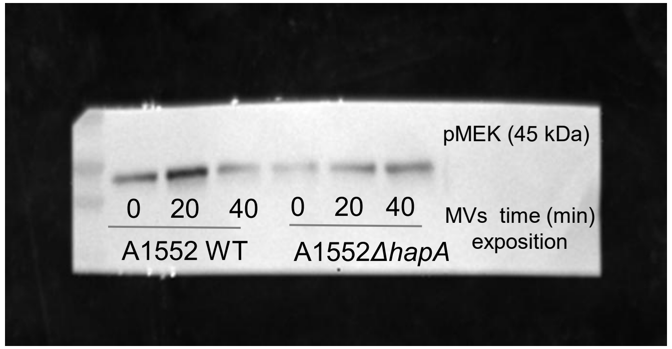

Piece 2

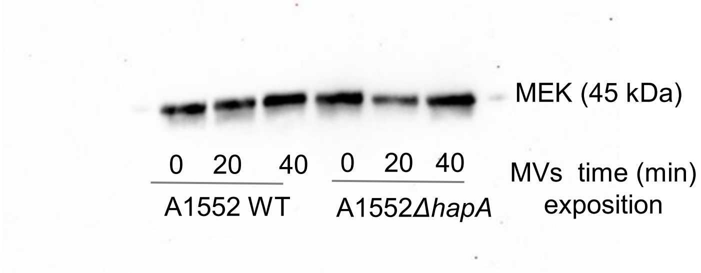

Begränsad delning

Uncropped membranes for Supplementary figure 6C. Membranes were cut into two parts prior to detection.

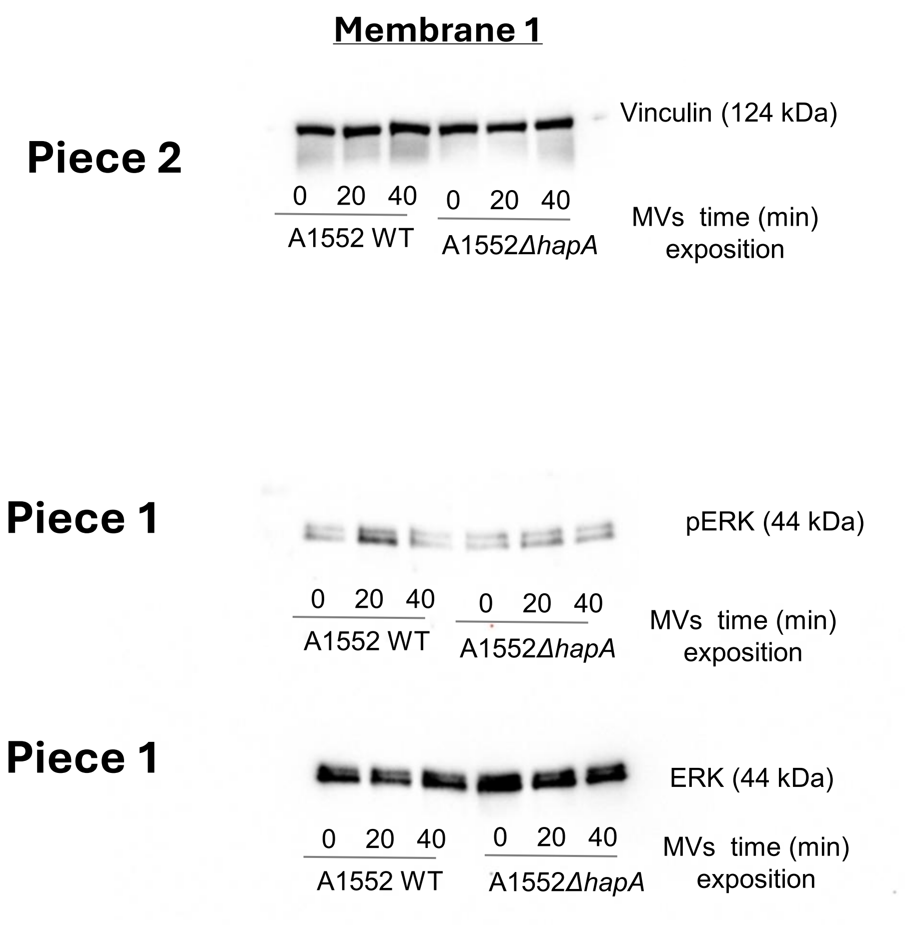

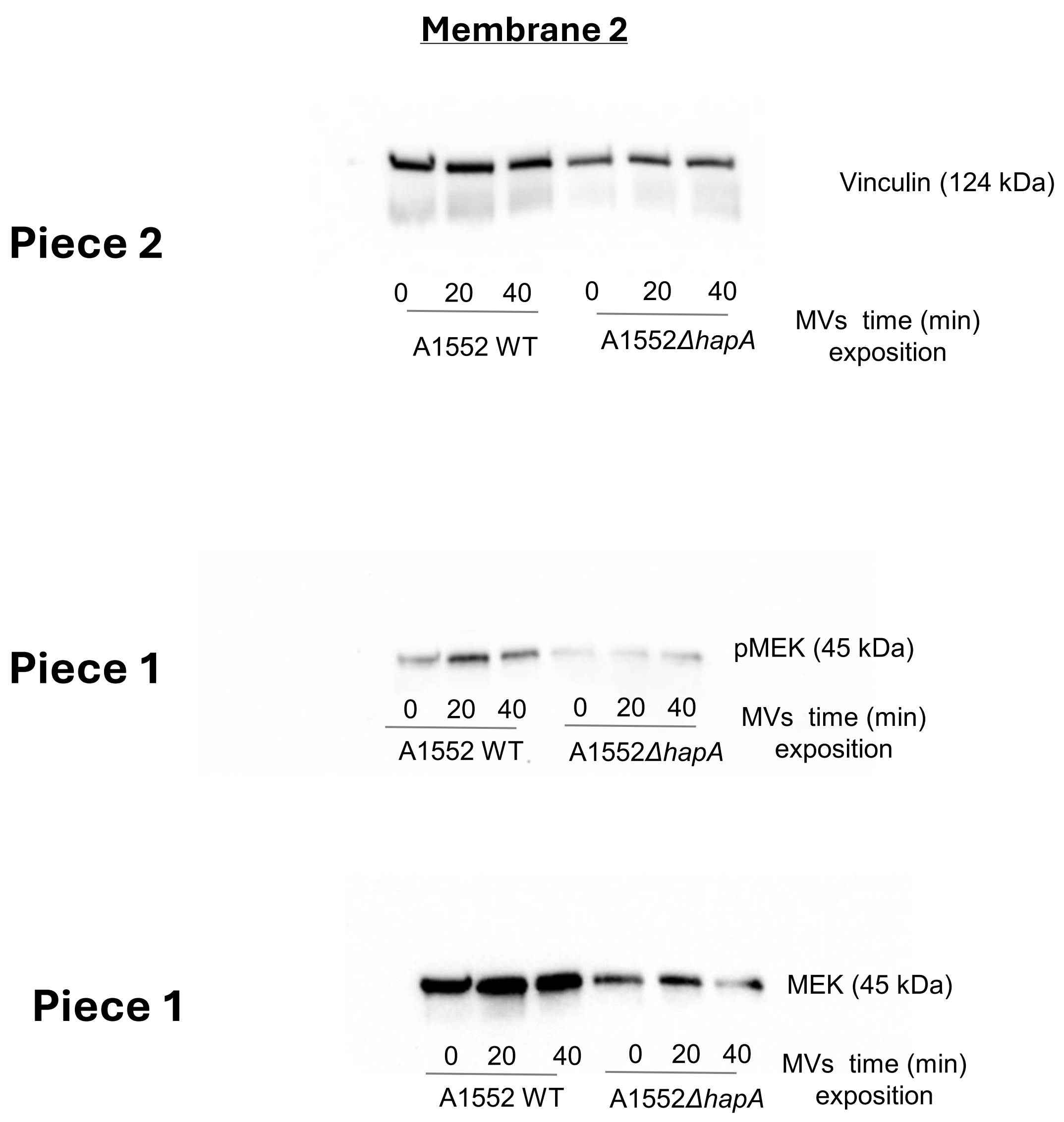

Begränsad delning

Uncropped membranes for Supplementary figure 7B . Membranes were cut into two parts prior to detection.

Piece 2

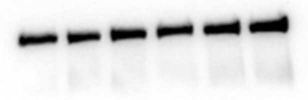

Piece 1

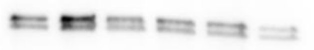

Piece 1

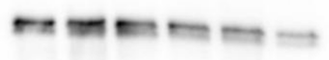

Uncropped membranes for Supplementary figure 7D . Membranes were cut into two parts prior to detection.

Piece 2

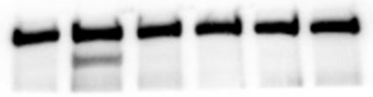

Piece 1

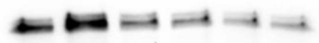

Piece 1

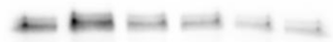

Begränsad delning

Uncropped membranes for Supplementary figure 8D . Membranes were cut into two parts prior to detection.

Piece 2

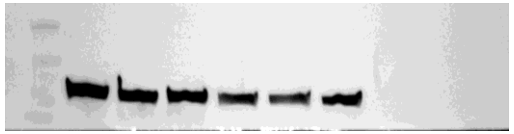

Piece 2

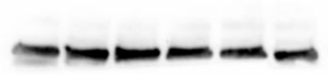

Piece 1

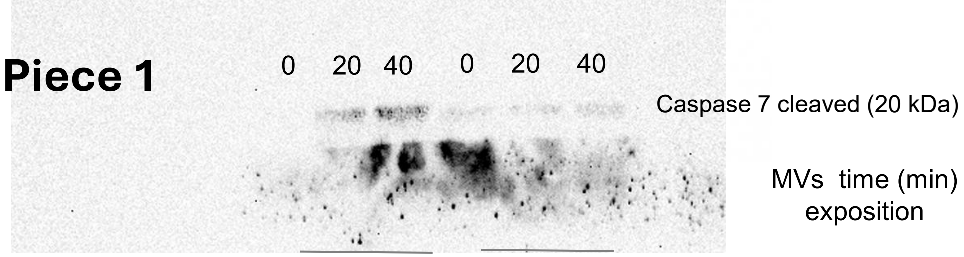

Uncropped membranes for Supplementary figure 8E . Membranes were cut into two parts prior to detection.

Piece 2

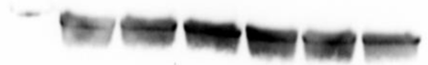

Piece 2

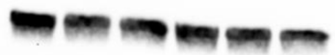

Piece 1

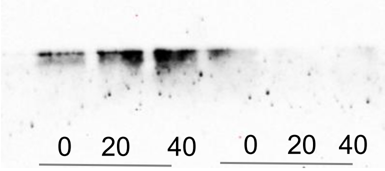

Supplement: Supplementary file 3 — Original Data [file 41420_2025_2691_MOESM3_ESM.pdf]
